# Advanced Stem Cell Therapy: 3D‐Bioprinted Brain‐Like Transplants for Alzheimer's Disease‐Like Dementia

**DOI:** 10.1002/advs.202510062

**Published:** 2025-11-07

**Authors:** Ke Gai, Yu Song, Dawen Gao, Qingning Nie, Xiao Luo, Caizhe Xu, Changhao Cai, Austin Smith, Xiang Li, Wei Shi, Lei Zhang, Wei Sun, Feng Lin

**Affiliations:** ^1^ Biomanufacturing Center Department of Mechanical Engineering Tsinghua University Beijing 100084 China; ^2^ Beijing Advanced Innovation Centre for Biomedical Engineering School of Engineering Medicine Beihang University Beijing 100191 China; ^3^ China Iron and Steel Research Institute Group Beijing 100081 China

**Keywords:** Alzheimer's disease, bioprinting, dementia, learning and memory, neural regeneration, stem cell therapy, transplantation

## Abstract

Alzheimer's disease (AD) is a neurodegenerative disorder that lacks effective treatments and urgently requires innovative therapeutic strategies. Although stem cell therapy has demonstrated efficacy in preclinical and clinical studies, it faces challenges such as low cell survival (<5%) and uncontrolled glial differentiation. This study aims to develop a 3D‐bioprinted neural patch to enhance stem cell therapy for AD. The hypothesis is that a supportive bioengineered microenvironment would improve cell integration and neuronal differentiation, leading to functional recovery. A tri‐component bioink (gelatin/alginate/fibrinogen) is created with tunable printability, biocompatibility, and biodegradation, establishing functional transplantation microenvironments for a 3D‐printed human induced pluripotent stem cell (hiPSC)‐derived neural progenitor cell (NPC) construct as a hippocampal patch. The system (TTBT) maintains NPC survival and promotes neuronal differentiation, neurite development, and calcium signaling in vitro. In AD‐like rats, these constructs improved cell retention (3.41‐fold over suspensions), enhanced neuron (79.21 ± 6.67% vs 65.08 ± 7.14%) and GABAergic neuron (29.85 ± 7.69% vs 15.93 ± 10.33%) differentiation, and restored long‐term potentiation (LTP) to 97.89% ± 19.84% of healthy control levels. Behavioral tests also show memory improvement, particularly in the Morris water maze. This 3D‐printed therapy not only holds potential for enhancing stem cell treatments but also addresses other 3D brain defects.

## Graphical abstract

A tri‐component biomimetic bioink(gelatin/alginate/fibrinogen) is developed to protect cells in critical pathological environments in AD. Using this bioink, a 3D‐printed NPC construct optimized for hippocampal transplantation is provided to improve cell retention and differentiation in an AD‐like model. This approach enhances memory recovery and offers a promising strategy for addressing neurodegenerative diseases through advanced bioengineered therapies.

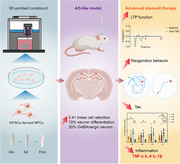

## 1 Introduction

Alzheimer's disease (AD) is an age‐related and devastating degenerative disease of the central nervous system, with approximately 95% of cases classified as sporadic forms lacking clear hereditary patterns. It is characterized by the formation of insoluble β‐amyloid (Aβ) plaques, hyperphosphorylation of tau proteins leading to neurofibrillary tangles (NFTs), and the resulting degeneration of neuronal cells and neuronal cell loss.^[^ ^1^ ^]^ Notably, functional impairment of neural cells has been identified as a direct contributor to cognitive decline. Emerging evidence suggests that GABAergic inhibitory interneuron dysfunction is associated with neuronal hyperexcitability, potentially exacerbating neuronal damage through this pathological cascade.^[^ ^2^, ^3^ ^]^ Current therapeutic strategies targeting these pathologies continue to face clinical trial failures, leaving AD without effective disease‐modifying treatments.^[^ ^4^, ^5^ ^]^ Stem cells, including neural stem cells (NSCs), can differentiate into functional neurons and glial cells,^[^ ^6^, ^7^ ^]^ demonstrating therapeutic promise through four interconnected mechanisms. First, NSC transplantation reduces pathogenetic Aβ accumulation and hyperphosphorylated tau‐induced NFT formation.^[^ ^8^, ^9^, ^10^ ^]^ Second, this approach suppresses neuroinflammation, which was recently proven to be a critical contributor to AD progression.^[^ ^11^, ^12^ ^]^ Third, paracrine signaling molecules like brain‐derived neurotrophic factor (BDNF) secreted by engrafted NSCs enhance endogenous neurogenesis and synaptic plasticity.^[^ ^9^, ^13^, ^14^ ^]^ Finally, emerging evidence demonstrates that transplanted NSCs can functionally restore lost neurons through differentiation, offering a direct therapeutic potential for AD.^[^ ^15^, ^16^ ^]^ This approach might be a promising therapeutic strategy for replenishing neurons with specialized functions, especially GABAergic interneurons. Despite their numerically sparse distribution, these interneurons exert essential neuromodulatory effects. Thus, GABAergic interneuron restoration could address the pathological hyperexcitability crucial for AD progression.

Current NSC transplantation therapies still face challenges, including low cell retention rates, uncontrollable differentiation directions, and difficulty repairing structural defects.^[^ ^17^ ^]^ Recently, 3D tissue engineering technologies have been investigated to address these issues. 3D organoids provide 3D structures containing complex neural cell types.^[^ ^18^, ^19^, ^20^ ^]^ Studies have shown that brain organoids can integrate into animal models of different ages through vascular and neural connections,^[^ ^21^, ^22^, ^23^ ^]^ demonstrating better defect repair than cell suspensions.^[^ ^24^ ^]^ However, clinical application of organoids remains limited by poor control over cell type distribution, mass production difficulties, and inconsistent size and shape.^[^ ^25^ ^]^ Bioprinting technology offers improved controllability and reproducibility compared to organoids. Researchers have developed protocols using ultra‐soft biocompatible materials to construct brain‐like structures.^[^ ^26^, ^27^, ^28^, ^29^ ^]^ Sensitive NSCs have been successfully printed in vitro and differentiated into functional neurons/glia within 3D constructs.^[^ ^30^, ^31^, ^32^, ^33^, ^34^ ^]^ By modulating extracellular matrix components or cell composition within bioinks, techniques such as acoustic assembly, coaxial printing, extrusion‐based bioprinting, and inkjet printing have been employed to construct Alzheimer's disease models, further demonstrating the ability of 3D bioprinting to provide cells with a more biomimetic microenvironment.^[^ ^33^, ^35^, ^36^, ^37^, ^38^, ^39^ ^]^ Furthermore, multilayered bioprinted brain tissues have achieved co‐culture and integration with mouse brain tissue.^[^ ^40^ ^]^ However, the AD brain microenvironment presents multifaceted pathological barriers, including altered mechanical properties, pathological protein aggregates, and chronic neuroinflammatory responses,^[^ ^41^ ^]^ that collectively compromise neural network reconstruction at lesion sites. This necessitates the development of tailored biomaterial systems specifically engineered to counteract AD‐specific pathophysiological challenges and enhance the neurogenic microenvironment for transplanted cells.^[^ ^42^, ^43^ ^]^ Previous studies utilizing nondegradable encapsulation materials for secretory cell transplantation demonstrated therapeutic effects in AD models and clinical trials, highlighting the role of controlled material degradation in mitigating immune responses.^[^ ^44^ ^]^ While degradable biomaterials (e.g., GelMA, hyaluronic acid, collagen, silk fibroin) have shown promise in stem cell transplantation for other neurological disorders,^[^ ^45^, ^46^, ^47^ ^]^ their application in AD remains unexplored. These findings demonstrate that biomaterials possessing 3D‐printability, neural stem cell‐maintaining properties, and immunologically harmonized degradation kinetics are critical for advancing stem cell‐based therapies. Transplantation systems engineered with such biomaterial frameworks exhibit breakthrough potential to enhance therapeutic outcomes in AD by sustaining stem cell functionality within pathological microenvironments.

**Figure 1:**
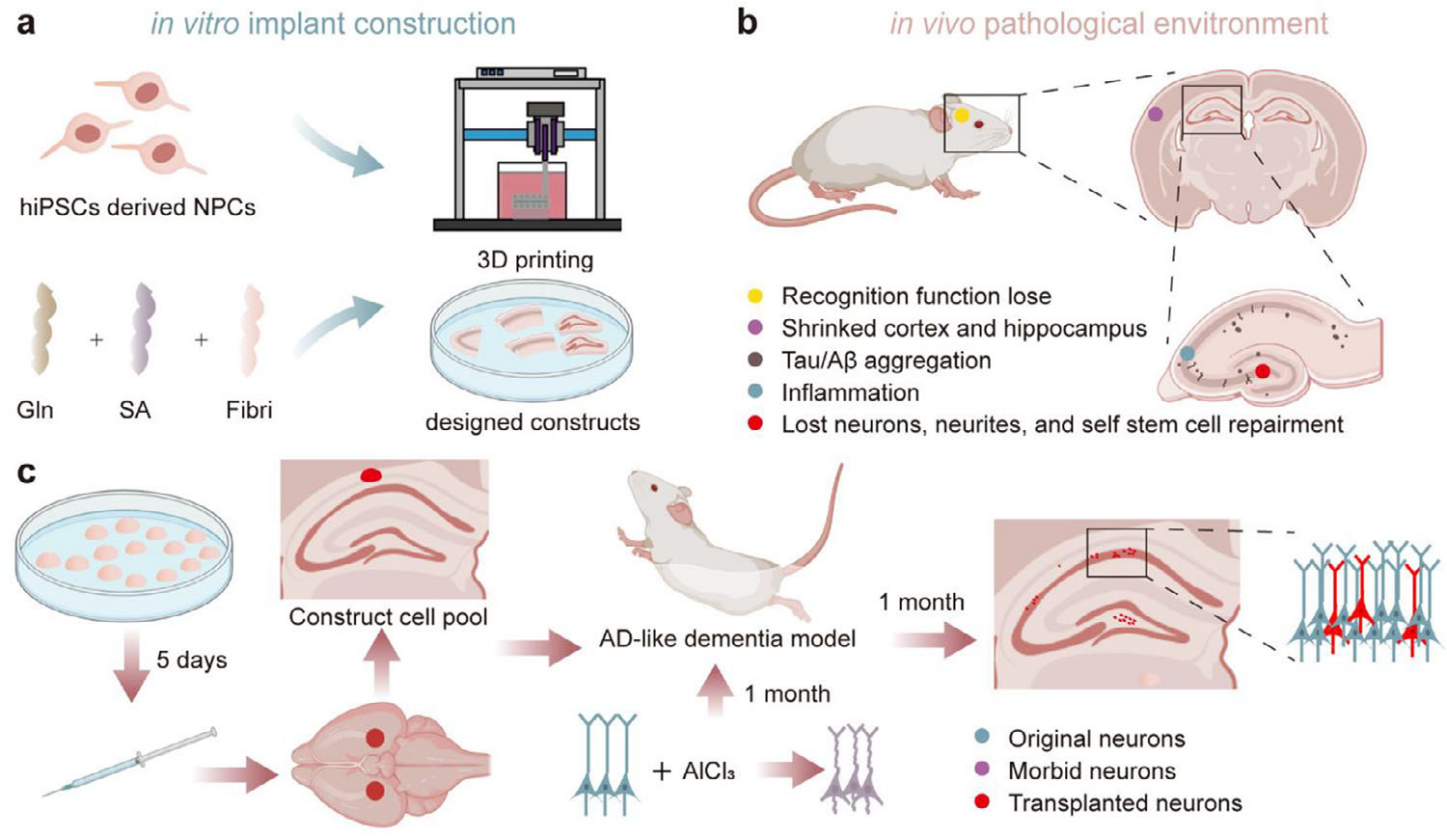
Design of 3D‐bioprinted neural constructs for AD therapy. a) In vitro fabrication of NPC constructs. Tri‐component hydrogel bioink (gelatin/ alginate/fibrinogen) was extruded into customized geometries using bioprinting. b) In vivo multiscale AD pathophysiology. The disease progression involves cognitive decline (behavioral level), global hippocampal atrophy (organ level), neuronal/synaptic degeneration with impaired endogenous repair (cellular level), and pathological microenvironment featuring neuroinflammation with Aβ/tau aggregation (molecular level). c) Therapeutic design of NPC construct transplantation. NPC constructs were transplanted into the CA1 hippocampus of AlCl_3_‐induced AD model rats on DAY 5 post‐printing to provide cell alternatives for cognitive recovery. Therapeutic effects were evaluated 1 month later. (Created with BioRender.com).

Therefore, this study proposed using 3D‐bioprinted neural progenitor cell (NPC) constructs with controlled geometry as transplants. These parahippocampal patches aim to establish stable cellular reservoirs for atrophic hippocampi, offering a novel therapeutic strategy for AD (**Figure** **1**). Building upon previous work,^[^ ^34^ ^]^ the gelatin/alginate/fibrinogen composite bioink mechanical properties, printability, chemical stability, degradation properties, and biocompatibility were optimized. The cross‐linked gelatin and alginate network provided cell‐adhesive motifs that enhanced the attachment and survival of grafted cells. The relatively stiff mechanical properties of the scaffold were designed to promote neuronal maturation within the softer AD brain tissue environment, while its slow degradation behavior offered sustained structural support, facilitating gradual cell migration and integration under inflammatory conditions. The printed NPC constructs were designed as 1‐mm‐diameter dot structures to accommodate rat brain size constraints. The constructs' dimensions were achieved through spiral‐path precision printing on a BIOMAKER4 (SP, China) bioprinter. The constructs maintained stemness, differentiation capacity, and fundamental function during a 14‐day in vitro culture. After optimization, DAY 5 post‐printed constructs were transplanted into the CA1 region of aluminum chloride (AlCl_3_)‐induced Alzheimer's disease‐like dementia (AD‐like) model rats. This rodent model recapitulates oxidative stress‐related features of Alzheimer's disease without altering the genetic background.^[^ ^48^ ^]^ Therapeutic outcomes were assessed via immunofluorescence (IF) staining, immunohistochemical (IHC) analysis, behavioral tests, and electrophysiology, investigating potential mechanisms, including pathological protein reduction, inflammation modulation, and controlled neural differentiation.

## 2 Results and Discussion

### 2.1 NPC Construct Design and Fabrication for AD‐Like Cognitive Recovery

This study developed 3D‐bioprinted NPC constructs to overcome neuronal loss and pathological microenvironmental barriers in AD‐like diseases. This was achieved through the design of: 1) neuron enrichment for functional repair, 2) host‐integratable macrostructure patterning, and 3) biomaterials providing biocompatible support. AD exhibits widespread neuronal degeneration and loss across multiple brain regions, with particular severity in hippocampal areas.^[^ ^49^ ^]^ This age‐related pathology might be associated with the dysfunction of NSCs.^[^ ^50^, ^51^ ^]^ Therefore, a designed transplant that could serve as a stem cell reservoir to replenish degenerated hippocampal neuronal populations during the degenerative disease process was proposed. Human cell compatibility with this therapy was investigated by strategically selecting human‐derived cellular components to systematically evaluate survival and functional integration in transplant construction and post‐engraftment pathological contexts. Human induced pluripotent stem cell(hiPSC)‐derived NPCs were selected as seed cells based on their capacity for controlled proliferation and multipotent neurogenic differentiation while mitigating immunogenic risks inherent to transplantation into patients. Distinct patterns of cell survival and neural network emergence were observed at low (3.57 × 10⁶ cells·mL^−1^), medium (6.67 × 10⁶ cells·mL^−1^), and high (2.67 × 10⁷ cells·mL^−1^) cell concentrations. Low cell concentrations supported viability but led to slow network formation. Medium cell concentrations promoted both good survival and rapid, mature network development. Lastly, high cell concentrations initially boosted survival but induced cell aggregation that disrupted network integrity and resulted in markedly reduced late‐stage viability (Figure S1, Supporting Information). After integrating outcomes related to cell behavior, printability, and clinical feasibility (including preparation time, cost, and printing stability), the medium concentration (6.67 × 10⁶ cells·mL^−1^) was selected for further study.

The pronounced hippocampal atrophy characteristic of AD^[^ ^52^ ^]^ created surgical space for extra‐hippocampal ventricular transplantation. While conventional organoid transplantation predominantly employs spherical geometries that establish linear tissue contact, this study developed a flat‐bottomed dot construct to achieve optimized surface contact with host hippocampal tissue. The design enhanced graft retention while reducing transplantation‐induced injury in animal models. Quantitative analysis of the CA1 transplantation site revealed curvature parameters critical for structural optimization: mean curvature 2.89 ± 1.39 × 10⁴ µm^−1^ (maximum 4.86 × 10⁴ µm^−1^). Host‐graft congruence was evaluated through dual mechanical criteria: 1) arc‐chord length discrepancy (*ϵ_rel_* = |(*s*‐*c*)/*s*| ≈ θ^2^/24) and 2) chord height‐to‐radius ratio (*δ* = *h/r* ≈ θ^2^/8). Although engineering standards typically enforce <1% tolerance, this threshold was extended to 5% to accommodate the shape adaptability of the ultra‐soft hydrogel material. Through small‐angle approximations, these constraints yielded θ_max_ ≈ 0.63 rad (36.1°). At maximal curvature (*r* = 2059.38 µm), this corresponds to an arc length of 1297.41 µm, dictating a 1‐mm base diameter to maintain <5% geometric mismatch (Figure S2a, Supporting Information). Additional complex architectures were fabricated to validate manufacturing capabilities (Figure S2b–d, Supporting Information).

**Figure 2:**
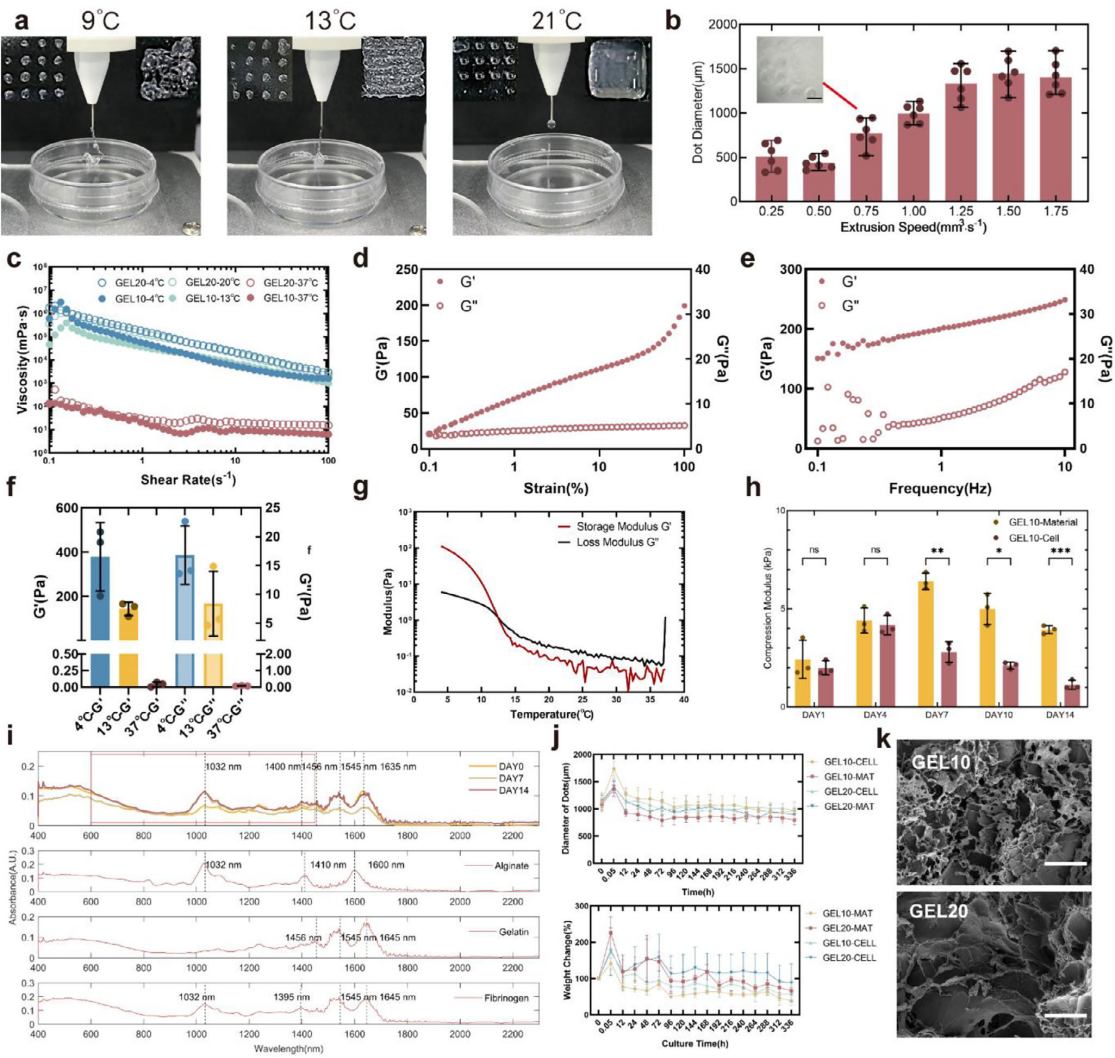
Bioink characterization for transplantable neural constructs. a) Temperature‐dependent printability of dot and grid constructs (4–21 °C). b) Extrusion speed optimization at 13 °C. 1 mm^3^·s^−1^ selected as optimal. Scale bar: 1 mm. (*n* = 6) c) Shear‐thinning behavior across tested temperatures. (representative of *n* = 3) d) e) Amplitude and frequency sweeps of GEL10 bioink at 13 °C. (representative of *n* = 3) f) Shear moduli under different temperatures (*n* = 3). g) Temperature sweep of GEL10 bioink. Gelation transition point at 13 °C. (G′/G″ crossover; representative of *n* = 3) h) Compressive modulus during 14‐day culture (*n* = 3). Data were analyzed with multiple unpaired *t*‐test. *p < 0.05, **p < 0.01, ***p < 0.001, and ****p < 0.0001. i) FTIR spectra confirming compositional stability in GEL10 cell‐laden constructs over 2 weeks (representative of *n* = 3). j) Structural fidelity and degradation post‐printing (*n* = 4). k) Scanning electron microscope (SEM) images of constructs. Scale bars: 50 µm. Data presented as mean ± SD.

From the material perspective, the transplants required mechanical compatibility with brain tissue to sustain NPC proliferation/differentiation and provide cytoprotection, along with printability for customized 3D fabrication. A tri‐component bioink containing gelatin, sodium alginate, and fibrinogen was optimized based on a hydrogel system established in previous studies.^[^ ^27^, ^29^, ^34^ ^]^ Sodium alginate(0.14% w/v) was maintained for rapid crosslinking, while fibrinogen (0.57% w/v) preserved bioactive motifs for cell–matrix interactions. Gelatin concentration was reduced from 5.71% to 2.56% w/v to enhance biomechanical support, with the original group designated as GEL20 and the optimized group as GEL10. The printability of GEL20 was previously investigated.^[^ ^34^ ^]^ The GEL10 bioink had a fabrication window of 11 to 15 °C for printing mesh structures, as shown in **Figure** **2**a. Entanglement occurred at 9 °C, indicating over‐crosslinking, resulting in irregular distortion of the final grid. At 21 °C, under‐crosslinking prevented the formation of the grid. At 13 °C (Figure 2g) the material crosslinking yielded the best formation results. In contrast, the dot constructs had a broader fabrication window than the grid constructs, with successful formation occurring between 9 and 21 °C (Figure S2e, Supporting Information). However, when the material was in an under‐crosslinked state, calcium chloride dispersed the structure, preventing the maintenance of the construct. 13 °C was designated as the standardized printing temperature to balance printability with cell tolerance. Besides, with the increasing extrusion speed, the line widths and dot diameters gradually increased (Figure 2b; Figure S2f,g, Supporting Information). Based on shape fidelity and structural uniformity, 1 mm^3^·s^−1^ was selected as the optimal extrusion rate.

GEL20 and GEL10 exhibited pronounced shear‐thinning behavior (Figure 2c), crucial for cell protection during extrusion. Rheological characterization through amplitude and frequency sweeps at the crosslinking temperature revealed stable viscoelastic behavior with consistent dominance of storage modulus (*G′*) over loss modulus (*G″*) (Figure 2d,e; Figure S3e,g, Supporting Information), demonstrating robust stability under varied printing parameters. At 37 °C, both bioinks showed minimal modulus variations (Figure S3a,b, Supporting Information). However, at 4 °C, GEL20 and GEL10 exhibited yield behavior (Figure S3c,d, Supporting Information), suggesting potential mechanical damage to cells under over‐crosslinked conditions. Temperature‐dependent shear modulus analysis revealed that the ultra‐soft material properties of GEL 10 bioink (<1 kPa) showed reduced stiffness compared to the GEL20 bioink (Figure S3f, Supporting Information). Compression modulus testing demonstrated time‐dependent modulus evolution: materials initially stiffened then softened during culture, with cell‐laden constructs showing softening transitions (within 1–4 kPa range) that mirrored NPC mechanical development of the extracellular environment (ECM).^[^ ^53^, ^54^, ^55^, ^56^ ^]^ (Figure 2h) The GEL10 group maintained lower elastic moduli than GEL20 group with and without cells (Figure S3f,g, Supporting Information).

The degradation of dot constructs within two weeks was systematically investigated through Fourier transform infrared spectroscopy (FTIR) (Figure 2i). Characteristic absorption bands of sodium alginate were identified at 1032 cm^−1^ (─O─ stretching), 1408 cm^−1^ (asymmetric stretching vibrations of ─COOH), and 1600 cm^−1^ (symmetric ─COOH stretching). Spectral shift from 1645 to 1635 cm^−1^ confirmed sustained alginate integrity during a 14‐day culture. For fibrinogen and gelatin, overlapping amide II (1545 cm^−1^, N─H bending) and amide I (1653 cm^−1^, C═O stretching) bands generated composite peaks at 1545 and 1,640 cm^−1^ in blended hydrogels, with a distinctive gelatin signature at 1456 cm^−1^ and fibrinogen‐indicative 1400 cm^−1^ band shifts. Notably, stable peak relative intensities and positions across time points (Figure S4a–c, Supporting Information) demonstrated robust compositional stability. Mass loss quantification revealed accelerated degradation in GEL10 groups, with cell‐laden constructs retaining 56.94 ± 26.20% initial mass at DAY 14 (Figure 2j). Linear regression analysis predicted complete degradation within one month (491.87–842.25 h, Figure S4d, Supporting Information). Diametric measurements showed well‐maintained structural fidelity (966.07 ± 162.26 µm final diameter) despite mass loss, confirming shape preservation throughout degradation. Comparable microstructural porosity was maintained between optimized and original formulations (Figure 2k). Comparisons across time points revealed enhanced surface roughness in cell‐laden constructs versus acellular counterparts (Figure S5, Supporting Information). These findings suggested that material optimization preserved matrix mesh density critical for cellular accommodation and cell–material interactions, likely promoted micro surface structures.

In summary, the NPC constructs were engineered via coordinated cellular, structural, and material optimization tailored for AD pathophysiology. The construct used hNPCs as seed cells to assess human viability and was designed as a 1‐mm‐diameter flat‐bottomed dot to match hippocampal morphology. The bioink (2.56% gelatin, 0.14% alginate, 0.57% fibrinogen) formed stably and mimicked brain tissue mechanics. The designs showed potential for improving transplantation in AD environments.

### 2.2 Functional Validation of Construct Development In Vitro

**Figure 3:**
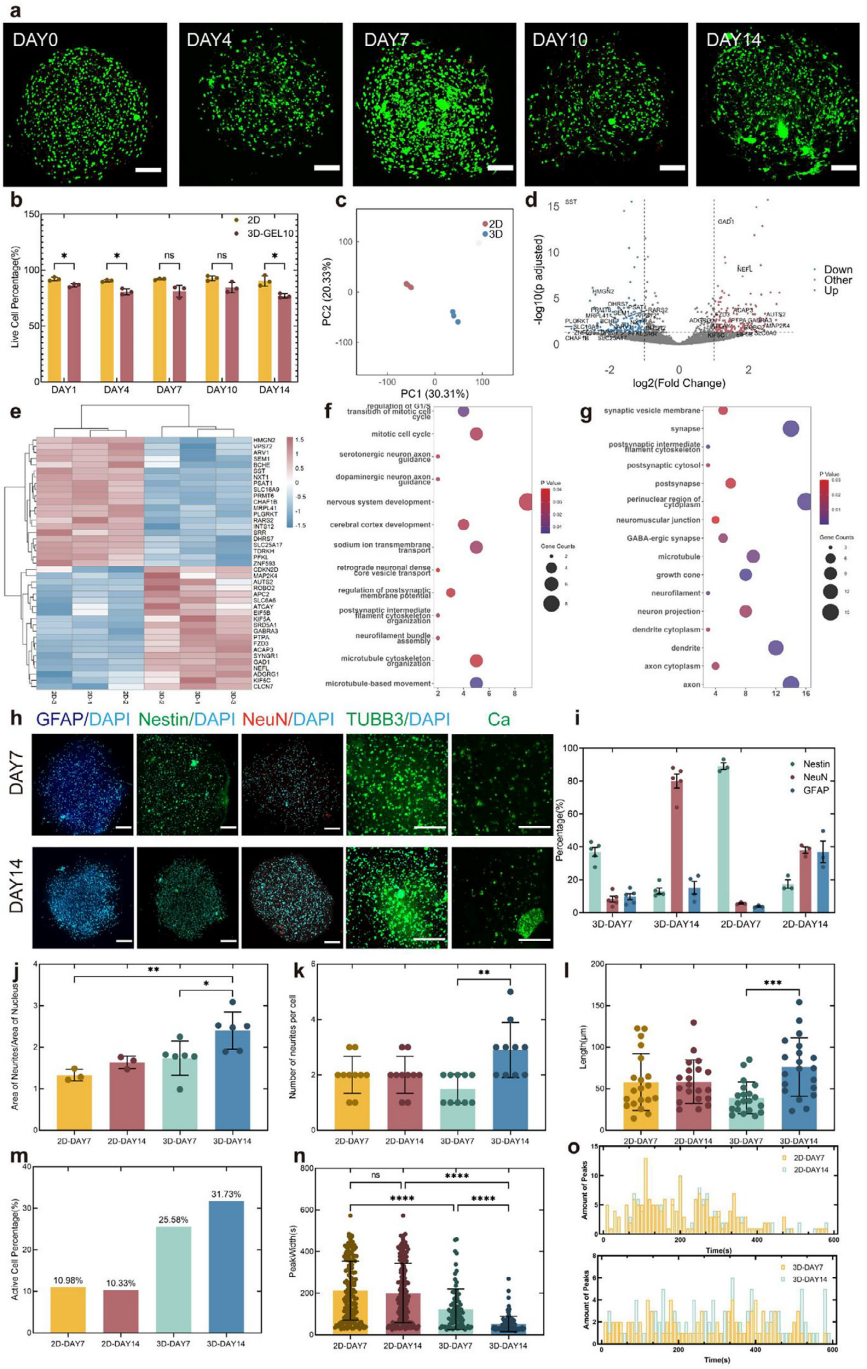
In vitro functional characterization of constructs. a) Viability assessment of construct‐embedded cells during 14‐day culture. Scale bar: 200 µm. b) Quantitative survival analysis (*n* = 3). Data were analyzed with multiple unpaired *t*‐test. c) Principal component analysis of RNA‐seq data comparing the cells in the 3D constructs with those in the 2D cultures on DAY 7 (*n* = 3). d) Differential gene expression analysis between the 3D and the 2D conditions (p_adj_ < 0.05, |log_2_FC|> 1). e) Heatmap of significantly different genes. f,g) GO‐BP and KEGG pathway enrichment of differentially expressed genes. h) IF staining for GFAP, Nestin, NeuN, TUBB3, and calcium (Fluo 4‐AM) on days 7 and 14. Scale bars: 200 µm. i) Differentiation quantification (3D: *n* = 5; 2D: *n* = 3). j–l) Synaptic area (3D: *n* = 6; 2D: *n* = 3), neurite number per cell (*n* = 10), and neurite length (*n* = 20) analyses. Synaptic area and length were analyzed by one‐way ANOVA with Tukey's multiple comparisons, and neurite counts by Kruskal–Wallis tests with Dunn's multiple comparisons. m–o) Calcium‐active cell proportion, signal duration, and peak timepoint analysis. Data were analyzed by Kruskal–Wallis tests with Dunn's multiple comparisons. (*n* = 169, 187, 88, 116). Data presented as mean ± SD. *p < 0.05, **p < 0.01, ***p < 0.001, and ****p < 0.0001.

The bioprinted constructs were functionally assessed during a 14‐day in vitro culture to determine optimal transplantation timing. Compared to the 2D cultures, initial 3D constructs using the GEL20 bioink exhibited reduced cellular viability, with mCherry‐transfected cells showing severe death (**Figure** **3**a,b; Figure S6a, Supporting Information). The optimized material system (GEL10) significantly improved survival rates, demonstrating 86.38 ± 1.25% viability post‐printing that stabilized to 76.99 ± 1.59% by DAY 14 (Figure S6b–d, Supporting Information). Live/dead imaging revealed progressive cell spreading from DAY 4 onward, with neurite‐like extensions spanning by DAY 14, indicating permissive microenvironments for neural network formation.

Transcriptomic analysis of cells harvested on DAY 7 from both 3D constructs and 2D cultures was performed to examine the impact of the 3D environment on differentiation. Principal component analysis revealed distinct clustering patterns between the two groups (Figure 3c), with 174 upregulated and 150 downregulated genes showing significant differential expression (|log2FC| > 1, adjusted *p* < 0.05) in 3D‐cultured cells (Figure 3d,e; Figure S6e,f, Supporting Information). Gene Ontology (GO) enrichment analysis demonstrated cell cycle‐related upregulation (regulation of G1/S transition of mitotic cell cycle, *p* = 1.02 × 10^−2^; mitotic cell cycle, *p* = 2.74 × 10^−2^) in 3D environments (Figure 3f), correlating with observed proliferative deceleration and indicative of differentiation initiation. Biological process terms were dominated by nervous system development (*p* = 3.20 × 10^−2^) and cerebral cortex development (*p* = 2.89 × 10^−2^). Upregulation of axon guidance (dopaminergic neuron axon guidance, *p* = 2.47 × 10^−2^; serotonergic neuron axon guidance, *p* = 3.28 × 10^−2^), sodium transmembrane transport (*p* = 2.26 × 10^−2^), and postsynaptic membrane potential modulation (*p* = 3.58 × 10^−2^) pathways (Figure 3f) indicated improvement of functions related to synapses. Cellular component analysis revealed axon (*p* = 7.52 × 10^−6^), synapse (*p* = 4.52 × 10^−4^), and dendrite (*p* = 1.25 × 10^−3^) enrichment (Figure 3g), supporting the previous hypothesis. Molecular function terms highlighted protein binding (*p* = 4.59 × 10^−10^) and microtubule motor activity (*p* = 1.66 × 10^−3^), suggesting interaction with materials and cytoskeletal remodeling for neurite extension. Kyoto Encyclopedia of genes and genomes (KEGG) analysis, a pathway enrichment‐based bioinformatics approach, showed GABAergic synapse (*p* = 8.78 × 10^−3^) pathway enrichment (Figure S6h, Supporting Information), providing potential specific differentiation direction in subsequent in vivo studies, which might not be mature in short in vitro culture.

Protein expression was measured through IF staining to examine the proportion of cells differentiating into different directions and neural network formation within the structure under in vitro culture conditions (Figure 3h). Under stemness maintenance conditions, the 2D cultures preserved higher NPC marker expression compared to 3D (Nestin+ 89.18 ± 3.59% vs 37.00 ± 6.03% in 3D, Figure 3i), potentially due to restricted growth factor accessibility in constructs. Remarkably, the 3D constructs maintained stable progenitor cells with minimal spontaneous differentiation (<10% GFAP+/NeuN+). Following stemness factor withdrawal, the 3D environments drove preferential neuronal differentiation (NeuN+ 80.09 ± 9.47% vs 38.05 ± 3.41% in 2D) while suppressing astrogliogenesis (GFAP+ 15.24 ± 7.56% vs 37.02 ± 11.38%, Figure S6i, Supporting Information). This suggested that under self‐differentiation conditions, the proportion of cells differentiating toward neurons was significantly higher in the 3D constructs compared to the 2D cultures, and was more conducive to replenishing damaged neurons in AD lesion areas.

Neurites were labeled with TUBB3 to investigate the morphology and connections of neurons. The findings revealed a higher ratio of neurite to nuclear area in the 3D constructs compared to the 2D cultures, which further increased upon the initiation of self‐differentiation (Figure 3j). This indicated an increased rate of occurrence and extension of neurites in the 3D constructs compared to 2D cultures. Additionally, the rise in the number of neurites per cell and the increase in neurite length within the 3D structures further demonstrated the enhanced complexity of the neural network (Figure 3k,l). A similar trend was not observed in the 2D cultures.

Furthermore, the Fluo 4‐AM calcium ion probe was employed to mark cellular calcium activity, and a labeled analysis of the cellular regions was conducted (Figure S6j, Supporting Information). The results showed a significantly higher proportion of active cells in the 3D constructs compared to the 2D cultures, with narrower calcium signal peak widths. These differences were notably pronounced within the first week of self‐differentiation. This suggested a significantly higher level of activity in the cellular signaling network and more efficient signal transmission within the 3D constructs than in the 2D cultures. The 2D cultures exhibited higher levels of signal intensity than the 3D cultures, with no trend of change over time (Figure S6k, Supporting Information), which was attributed to the light obstruction caused by the 3D hydrogel material during imaging. Surprisingly, a globally rhythmic calcium signal was observed within the 3D constructs, with an oscillation period of approximately 60 s, as shown in Figure 3o. This rhythmic pattern was not observed in the 2D cultures or the previous grid structures^[^ ^34^ ^]^ but is seen in more mature primary neural structures.^[^ ^29^ ^]^ The observed dot‐shaped geometry of the constructs appeared to correlate with enhanced functional signaling networks among the encapsulated cells. This was further supported by live/dead staining, which indicated earlier neurite outgrowth in cells located toward the center of the structures. These preliminary findings suggested that structural tension during dot formation may influence cellular behavior. The precise mechanistic relationship between construct geometry and calcium signaling rhythms remains to be fully elucidated. Further experimental investigation, such as quantitative biomechanical modeling or targeted perturbation studies, would be valuable to clarify the underlying mechanism.

When comparing the development of cells in the 3D constructs to those in the 2D cultures in vitro, the cells in the constructs exhibited a higher proportion of neuronal differentiation and a more mature foundation for neural network formation. Guided by AD's pathophysiology requiring both neuronal replenishment and neural progenitor restoration, DAY 5 constructs were selected for transplantation, balancing the stability of cell extension in the structures with the maintenance of stemness.

### 2.3 Cell Retention and Neuronal Differentiation in the AD‐Like Model

**Figure 4:**
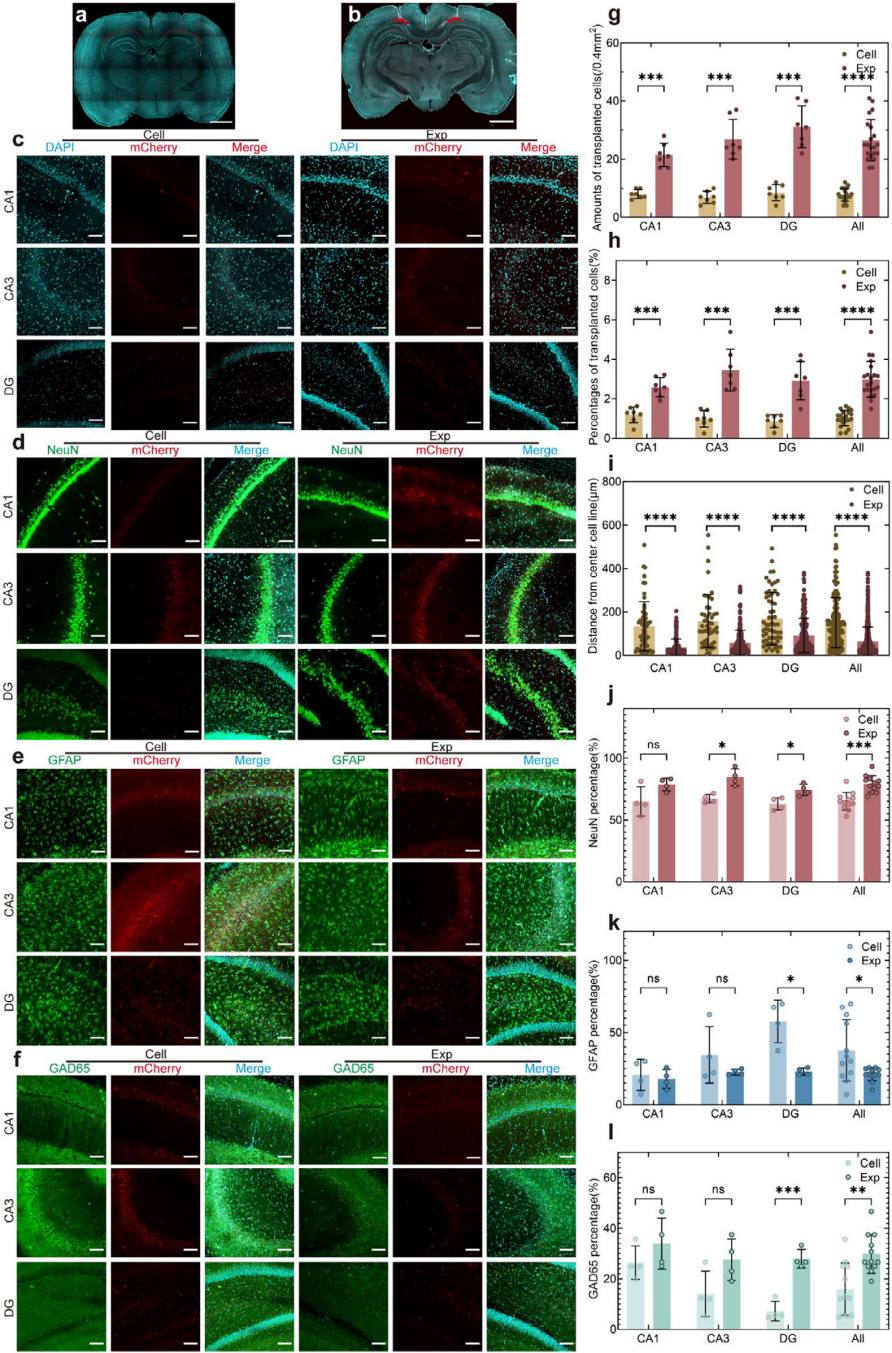
Cell retention and differentiation of construct and cell suspension transplantation strategies. a) Schematic of cell suspension injection, identified as Cell (red: injected cells). b) Stem cell construct transplantation, identified as Exp (red: transplantable construct). c) Spatial distribution of retained cells in the hippocampal subregions ≥1 month post‐transplantation. d–f) Co‐localization of transplanted cells with NeuN (neurons), GFAP (astrocytes), and GAD65 (GABAergic neurons) across the hippocampi. g–i) Quantification of the retained cell counts (*n*=7, 7, 7, 21), engraftment ratios (*n*=7, 7, 7, 21), and proximity to principal cell layers (*n*=59, 48, 59, 179, 219, 223, 276, 718). j–l) Differentiation rates toward neurons, astrocytes, and GABAergic neurons (*n*=4, 4, 4, 12). Data presented as mean ± SD. For panels (g, h, j), and (l), multiple unpaired *t*‐tests were applied. Mann–Whitney tests were used for panel (i), and Welch's t tests were used for panel k. Scale bars: 2 mm (a, b); 100 µm (c–l). *p < 0.05, **p < 0.01, ***p < 0.001, and ****p < 0.0001.

An aluminum‐overload animal model mimicking key disease features through Aβ aggregation and oxidative stress pathways was established to investigate environmentally triggered AD‐like pathology,^[^ ^57^, ^58^, ^59^ ^]^ following validated protocols in Wistar rats.^[^ ^60^, ^61^ ^]^ Two therapeutic strategies, 3D construct transplantation (Exp) and conventional cell suspension injection (Cell) (**Figure** **4**a,b), were designed and systematically compared ≥1 month after transplantation.

Transplanted cells with mCherry within the field of view were quantified to assess cell retention (Figure 4c). The controls (non‐transplanted and empty material‐transplanted groups) were imaged under identical conditions to account for spontaneous tissue fluorescence (Figure S7a, Supporting Information). While faint red shadows and noise puncta were observed in principal cell layers, no mCherry‐positive signals matching cellular criteria (≥5 µm diameter, sub‐circular morphology) were detected. Transplanted cells were adjusted using these criteria, and all analyses utilized calibrated data. Using comparable initial cell numbers, the construct group showed extended cellular morphologies and retained 3.41‐fold more cells per unit area compared to the cell suspension group (Figure 4c,g). Total cell counts were estimated via DAPI‐stained nuclei, and transplant retention rates were calculated. The construct group exhibited higher retention rates, consistent across hippocampal subregions (Figure 4h). Retained cells in the construct group localized closer to principal cell layers (Figure 4i; Figure S7b, Supporting Information), suggesting enhanced neural circuit integration potential.

Further, IF staining of brain slices was conducted to observe the differentiation of retained cells (Figure 4d–f). Overall, the cells in the construct group showed a higher proportion of neuronal differentiation (79.21% ± 6.67%), a lower proportion of astrocyte differentiation (21.18% ± 4.52%), and lower viability compared to the cell suspension group (Figure 4j,k). The observed attenuation of the difference between groups (compared to in vitro findings) likely stems from accelerated hydrogel degradation in long‐term in vivo microenvironments, which progressively diminished structural confinement effects and improved microenvironmental convergence between groups. Notably, the cell suspension group exhibited higher neuronal differentiation (65.08 ± 7.14% neurons and 37.62 ± 21.25% astrocytes) compared to prior reports(19.35 ± 0.84% neurons and 40.25 ± 0.57% astrocytes^[^ ^62^ ^]^). This might derive from the hippocampal‐adjacent ventricular transplantation location, which caused selective attrition of unintegrated cells and activity‐dependent survival bias through cerebrospinal fluid clearance. Both the cerebral cortex and hippocampus contain two major types of neurons: glutamatergic neurons and GABAergic neurons,^[^ ^63^ ^]^ with the former comprising about 80–90%.^[^ ^2^ ^]^ Substantial neuronal degeneration and loss in AD underscored the therapeutic value of achieving high neuronal differentiation rates in transplanted constructs to replenish damaged neural circuits. In contrast, astrocytes underwent significant pathological alterations in AD, showing elevated proportions of reactive, disease‐associated astrocytes^[^ ^64^, ^65^ ^]^ that colocalize with Aβ deposits.^[^ ^66^ ^]^ Furthermore, surgical transplantation trauma risked inducing glial scarring that inhibited neuronal regeneration.^[^ ^67^ ^]^ Compared to conventional cell suspension approaches, the constructs in this study demonstrated a remarkable reduction in astrocyte differentiation, reducing potential glial scaring or increased astrocyte reactivity due to construct‐derived astrocytes. It was noteworthy that the limited quantity of transplanted cells constrained by the size of transplants resulted in insufficient cell density for analysis in some brain slices, potentially introducing bias into the statistical analysis.

Recent studies have shown that in AD, damage to interneurons leads to abnormal hippocampal excitation and dysfunction.^[^ ^68^, ^69^ ^]^ Hippocampal GABAergic cells are predominantly classified as interneurons.^[^ ^2^ ^]^ Although fewer in number, GABAergic neuron supplementation could play a crucial regulatory role in AD.^[^ ^68^ ^]^ Previous RNA sequencing revealed higher enrichment of GABAergic neuron‐related pathways in cells present in constructs. Therefore, GAD65 staining was applied to identify GABA neuron differentiation in vivo. Co‐localization staining showed that the construct group had a GABAergic neuron differentiation rate (29.85% ± 7.69%) more than double that of the cell suspension group (15.93% ± 10.33%) and was more homogenous.

In terms of the engraftment and differentiation of the transplanted cells, the 3D construct group demonstrated superior performance compared to the cell suspension group. The construct group exhibited higher retention rates with a distribution closer to the principal cell layers and a higher proportion of differentiation into neurons, particularly GABAergic neurons. The increased ratio of GABAergic neurons in the construct might establish a basis for enhancing therapeutic efficacy at reduced transplantation dosages, potentially addressing critical barriers in neural regeneration strategies for AD.

### 2.4 Construct Transplantation Restored Hippocampal Neural Networks and Ameliorated Pathological Hallmarks

A four‐group experimental design was implemented to evaluate transplantation outcomes: Ctrl (non‐modeled, saline injection), Mat (modeled with material constructs), Cell (modeled with cell suspension transplants), and Exp (modeled with cell constructs). The Ctrl group provided baseline neurophysiological parameters, while the Mat group represented untreated pathology controls. The Cell and the Exp groups were designed to assess conventional versus engineered therapeutic strategies, respectively. All analyses were performed ≥1 month post‐transplantation to assess sustained therapeutic effects.

**Figure 5:**
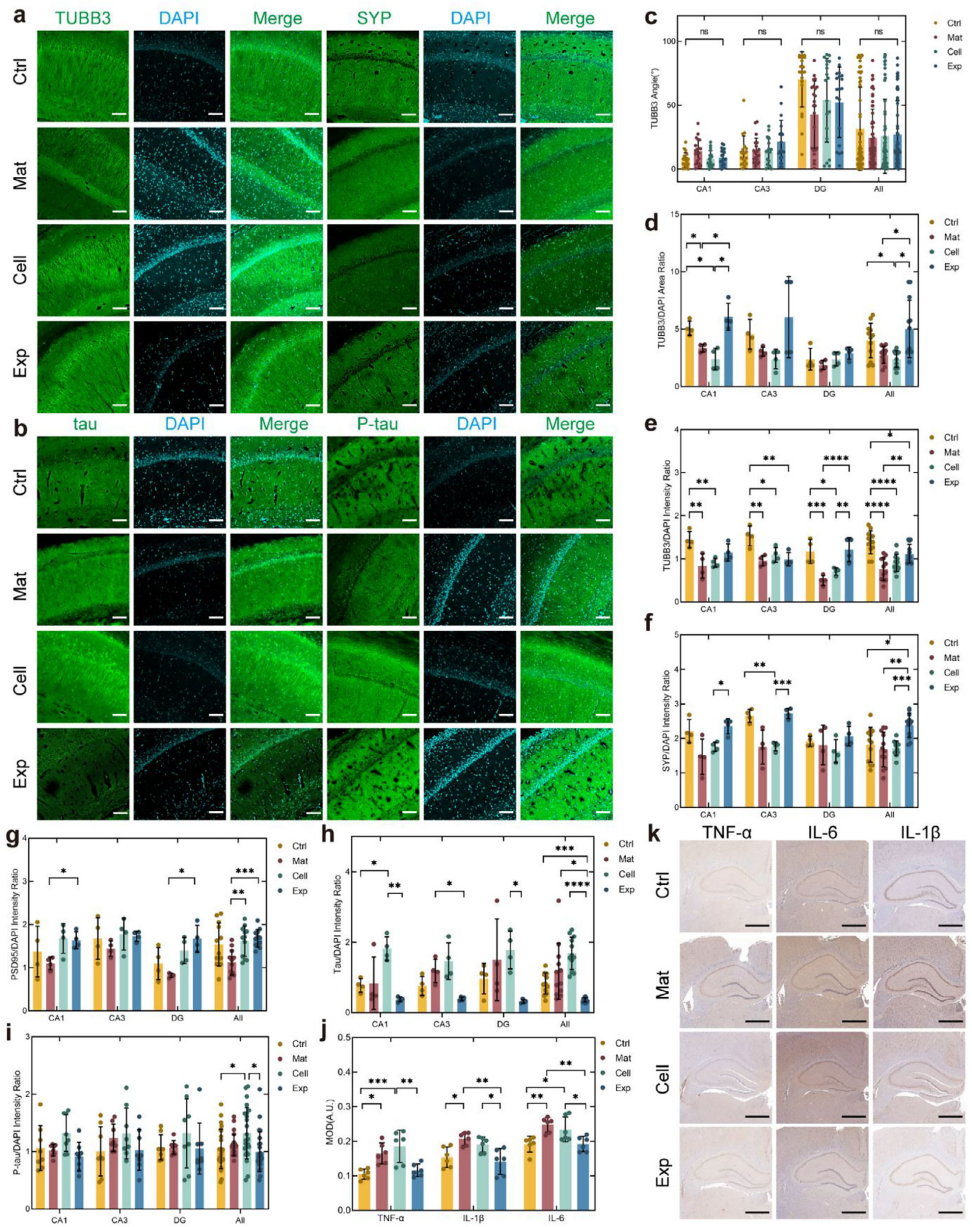
Hippocampal network restoration and pathological amelioration via construct transplantation. a) Immunofluorescence staining images of TUBB3 and SYP in hippocampal brain sections. b) Immunofluorescence staining images of tau and P‐tau in hippocampal brain sections. c) Analysis of the orientation of TUBB3‐labeled neurites near the principal cell layer. *n* = 19, 19, 21, 59, 20, 20, 23, 63, 18, 18, 19, 55, 20, 19, 19, 58. d,e) DAPI‐normalized statistical results of TUBB3‐positive area and fluorescence intensity. *n* = 4, 4, 4, 12. f–h) DAPI‐normalized statistical results of fluorescence intensity for SYP, PSD‐95, and tau. *n* = 4, 4, 4, 12. i) DAPI‐normalized statistical results of fluorescence intensity for P‐tau. *n* = 8, 8, 8, 24. j,k) IHC images and mean optical density statistical analysis of TNF‐α, IL‐1β, and IL‐6. *n* = 6. Data are presented as mean ± SD. For panels e and i, Two‐way ANOVA with Tukey's multiple comparisons test was applied. For panels d, f, g, and h, Two‐way Welch's ANOVA with Games–Howell post hoc tests was used. Panel c was analyzed using the Scheirer Ray Hare test followed by Dunn's multiple comparisons test, and panel j was analyzed using ordinary one‐way ANOVA with Tukey's multiple comparisons test. Scale bars: 100 µm (a,b), 1 mm (k). *p < 0.05, **p < 0.01, ***p < 0.001, and ****p < 0.0001.

The microtubule‐associated protein β‐tubulin‐III (TUBB3), a cytoskeletal marker critical for axonal guidance and neural development, was employed to map hippocampal neural network distribution (**Figure** **5**a; Figure S8a, Supporting Information). Orientation analysis of TUBB3‐positive neurites adjacent to principal cell layers revealed no significant difference across groups in CA1, CA3, or DG subregions (Figure 5c), indicating that transplanted cells maintained host‐like directional organization. Quantification of neurite complexity (TUBB3+/DAPI area ratio) demonstrated therapeutic advantages in CA1 region, where the Exp group outperformed both the Mat and the Cell groups, achieving levels comparable to the Ctrl group. This pattern persisted in whole‐hippocampus analyses, suggesting construct‐mediated neurite restoration in the subregion with organized axons (Figure 5d). Normalized fluorescence intensity analysis (DAPI‐corrected) revealed hierarchical TUBB3 expression across the groups: Ctrl > Exp > Cell > Mat in CA1, DG, and global hippocampus (Figure 5e), with CA3 showing exclusive Ctrl dominance. These findings demonstrated that construct transplantation improved neural cytoskeletal protein expression while surpassing suspension therapy in preserving neural architecture, with region‐specific efficacy correlating to native axonal organization. Raw fluorescence data and normalization protocols were detailed in Figure S8b–d (Supporting Information) and methods.

Hippocampal neural network function in learning and memory critically depends on synaptic plasticity. IF analysis was performed to evaluate construct‐mediated synaptic restoration using presynaptic marker synaptophysin (SYP) and postsynaptic density protein 95 (PSD‐95) (Figure 5a; Figure S8a, Supporting Information). Quantitative SYP intensity analysis demonstrated comparable presynaptic density between Ctrl and Exp groups across CA1, CA3, and DG subregions, with both groups surpassing the Mat and the Cell groups. Notably, the Exp group exhibited significantly higher SYP expression levels than the Ctrl group in whole‐hippocampus assessments. Parallel analysis of PSD‐95 revealed similar therapeutic benefits, though the Cell group demonstrated unexpectedly high expression levels. This synaptic enhancement might be attributed to neurotrophic paracrine signaling from transplanted stem cells,^[^ ^13^ ^]^ while xenogeneic transplantation effects could contribute to aberrant PSD‐95 elevation in the Cell group.

The high expression and aggregation of tau protein and its phosphorylated product, phospho‐tau (P‐tau), are widely recognized as significant pathological hallmarks of AD. The IF analysis revealed construct‐mediated tauopathy mitigation. Tau expression was significantly reduced in the Exp group compared to the Mat and the Cell groups (Figure 5b,h). The original fluorescence intensity of tau was similar to the corrected fluorescence intensity results (Figure S9a,d, Supporting Information). Analysis of the corrected fluorescence intensity revealed that the P‐tau level in the Mat group was higher than that in the Ctrl group, although the difference was not statistically significant (Figure 5i). Surprisingly, the Cell group exhibited even higher P‐tau levels compared to the Mat group, and the level was significantly different from the Ctrl group. Based on the original fluorescence intensity data, both the Mat and the Cell groups showed significantly stronger signals than the Ctrl group. While the Exp group demonstrated significant improvement relative to the Mat group, it did not reach the level observed in the Ctrl group (Figure S9e, Supporting Information). However, Aβ deposition remained undetectable across all groups (Figure S10, Supporting Information), which may be attributed to the relatively young age of the animals and the specific methodology used for AD induction. Semi‐quantitative analysis of integral optical density (IOD), mean optical density (MOD), and positive area fraction did not reveal significant intergroup differences. A slight increase in positive area was observed in the Material (Mat) and Cell groups compared to the Control (Ctrl) and Experimental (Exp) groups. This subtle change could potentially indicate a modest reduction in soluble Aβ42 following construct transplantation; however, the differences were not statistically significant. Longitudinal studies extending beyond the current 1‐month observation period might be required to fully capture Aβ pathology progression.

Elevated levels of tau phosphorylation and the spreading of pathological tau^[^ ^70^ ^]^ are closely associated with neuroinflammation, another key pathological hallmark of Alzheimer's disease. IHC staining of TNF‐α, IL‐1β, and IL‐6 in brain tissue sections was performed, and comparative analyses based on MOD were conducted to evaluate neuroinflammatory responses (Figure 5j,k). Results showed higher TNF‐α expression in the Cell group than in the Mat group, though not significantly. IL‐1β and IL‐6 levels were comparable between the two groups. Analysis of all three inflammatory cytokines revealed that the Exp group reduced inflammation to levels comparable to the Ctrl group, indicating that construct transplant significantly alleviated native disease‐associated and transplantation‐induced inflammation. Furthermore, IF staining of GFAP across the whole tissue sections revealed that the modeling method induced a glial response, leading to increased GFAP expression (Figure S11, Supporting Information). Construct transplantation significantly reduced GFAP levels to an extent comparable to the Ctrl group. Although cell transplantation alone resulted in a slight, non‐significant reduction in the normalized fluorescence intensity of GFAP, an increase was observed specifically in the CA1 region adjacent to the transplantation site. This phenomenon was potentially associated with the high proportion of GFAP‐positive differentiated transplant‐derived cells. Analysis of the raw fluorescence intensity showed a non‐significant elevation in the Cell group compared to the Mat group. Therefore, although the implantation of a pure cell suspension itself exhibited a high proportion of astrocyte differentiation, leading to locally elevated GFAP levels in the CA1 region, it did not significantly increase the overall GFAP expression level in the tissue. Thus, the astrocytic response elicited by cell transplantation alone was comparable to that induced by the material transplantation group. Under AD‐like inflammatory conditions, resident microglia become activated, which can be labeled with CD68. IHC results revealed a higher presence of CD68‐positive activated microglia in both the Mat and the Cell groups, with notable morphological differences (Figure S12, Supporting Information). The microglia in the Mat group exhibited a more contracted shape with shorter, thicker processes, while those in the Cell group, though also amoeboid, appeared more extended and showed lower immunostaining intensity. In comparison, the Ctrl and Exp groups displayed only minimal and scattered CD68‐positive signals. Although no statistically significant differences were observed in MOD across groups, IOD analysis indicated higher CD68 expression in the Mat group compared to the Ctrl group, while the Cell group differed from the Ctrl group in terms of positive area. Moreover, elevated CD68‐positive signals were observed at the transplantation scar in the Mat and Cell groups, suggesting that the implantation procedure itself induced a certain inflammatory reaction. CD3‐positive T cells were observed in the hippocampus of the Mat group, with sporadic presence also noted in the Cell group, whereas no such cells were detected in the Ctrl or Exp groups (Figure S13, Supporting Information). This indicated a marked inflammatory response in the model group, which recruited a small number of T cells. Cell transplantation appeared to alleviate this response, with a more substantial reduction seen in the construct transplantation group. Semi‐quantitative results were generally consistent with morphological analysis, though the Cell group exhibited moderately higher MOD, IOD, and positive area fraction compared to the other groups. Moreover, CD3‐positive cells were present at the scar across all groups that underwent the transplantation procedure, consistent with CD68 findings and indicative of surgery‐related inflammation. Overall, in line with the trends observed for TNF‐α, IL‐6, and IL‐1β, the CD68 and CD3 results suggested that construct transplantation alleviated inflammation in the AD‐like model, while also highlighting the inflammatory effects attributable to the implantation process itself.

### 2.5 Construct Transplantation Improved Cognitive Function in AD‐Like Model Animals

**Figure 6:**
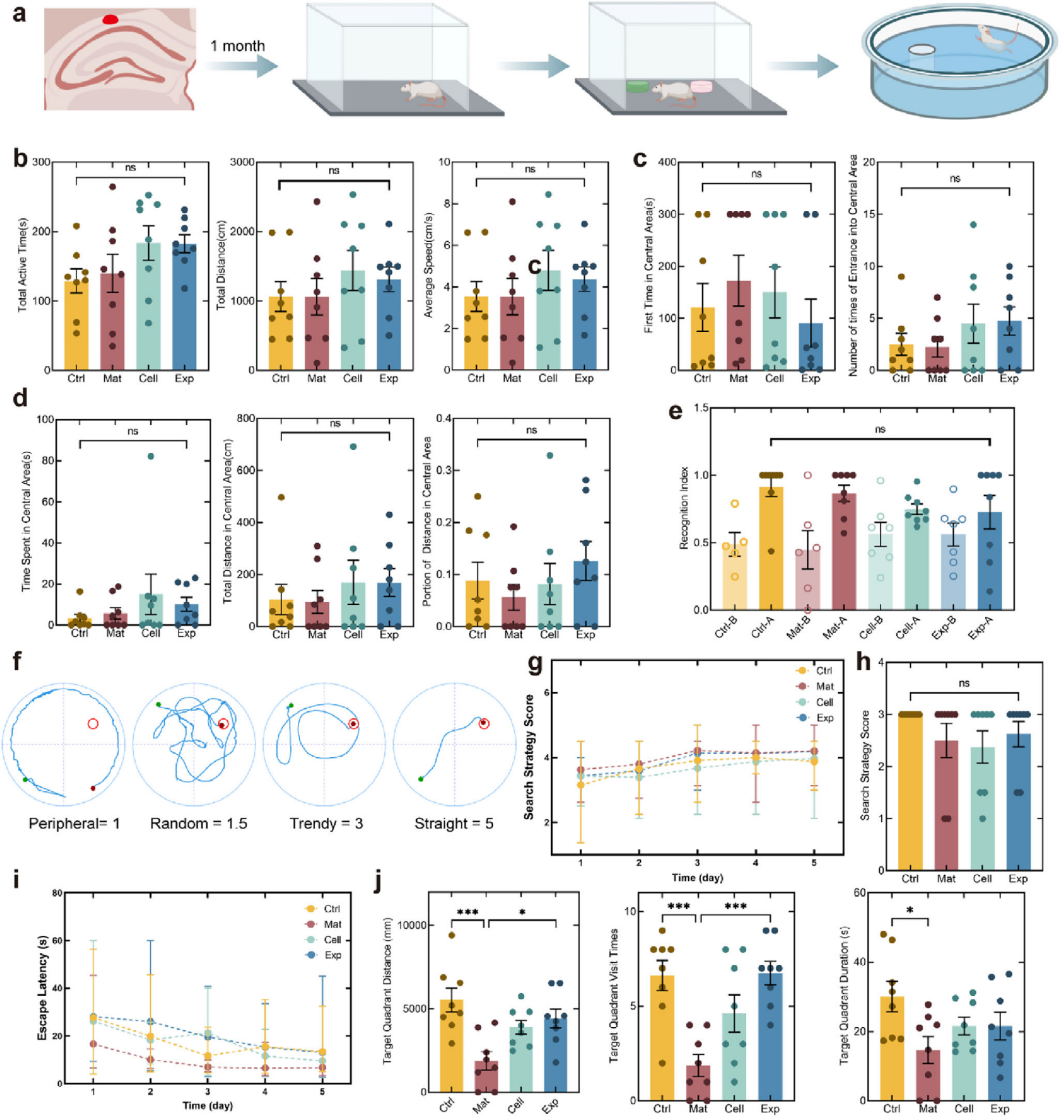
Cognitive functional restoration through 3D construct transplantation in AD‐like model rats. a) Schematic of behavioral assessment timeline, including open field, novel object recognition, and Morris water maze tests conducted at ≥1 month post‐transplantation. b–d) Open field analyses: Total active time, distance, and velocity (b); First time in the central area (300 s for no entry) and the number of entries (c); Time, distance in the central area, and distance ratio(d). e) Novel object recognition performance quantified by recognition index (novel/total exploration time ratio). f) Example of four search strategies (peripheral, random, trendy, straight) and respective scores in water maze testing. g,h) Morris water maze acquisition training progression and final test score performance. i) Escape latency reduction during training days. j) Target quadrant distance, visit times, and duration during probe trials. *n* = 8. Training data presented as mean ± range; other data presented as mean ± SEM. For panel (b, j), and the second analysis plot of panel c) Ordinary one‐way ANOVA with Tukey's multiple comparisons test was applied. For panels (d, e, h), and the first analysis plot of panel (c), Kruskal–Wallis test with Dunn's multiple comparisons test was used. *p < 0.05, **p < 0.01, ***p < 0.001, and ****p < 0.0001.

Open field test, novel object recognition, and Morris water maze tests were conducted at ≥1 month post‐transplantation to evaluate therapeutic efficacy at the behavioral level (**Figure** **6**a).

The open‐field test is utilized to evaluate exploratory behaviors, anxiety, and depressive‐like behaviors in a novel environment by monitoring the activities of animals in an open space. Rats modeled for AD might exhibit reduced time spent in the central area, indicative of heightened anxiety, and a decrease in overall activity distance, suggesting depressive‐like behavior. After model establishment, a comparative analysis between the control and the model groups revealed that the model group showed lower total distance, central area distance, central area time, and central area distance ratio than the control group. However, these differences were not statistically significant, suggesting that the AD‐like model did not exhibit pronounced anxiety or depressive behaviors (Figure S14a–d, Supporting Information). Similarly, in the comparative analysis of results post‐transplantation, no statistically significant differences were observed (Figure 6b–d). The time to first enter the central area followed the order Exp < Ctrl < Cell < Mat, and correspondingly, the ratio of central area activity distance followed the order Exp > Ctrl > Cell > Mat. However, no consistent trends were observed for the activity time and the total distance in the central area.

The novel object recognition test was employed to further evaluate changes in short‐term learning and memory. The test used the ratio of time spent exploring the new object to the total exploration time as the Recognition Index. Following model establishment, the model rats exhibited a significantly lower Recognition Index compared to the control group, indicating impaired short‐term memory and learning abilities (Figure S14e, Supporting Information). However, in the post‐transplantation tests, all four groups of animals showed a clear preference for the novel object (Figure 6e). This suggests that the impaired preference for novel objects in the model might be an acute manifestation rather than a symptom of a degenerative disease, as their recognition ability naturally recovered after one month of normal housing.

The Morris water maze, a gold‐standard paradigm for assessing spatial learning and memory in rodents,^[^ ^71^ ^]^ revealed significant cognitive restoration through construct transplantation (Figure 6f). Following the 5‐day training, model animals were tested for spatial memory on DAY 6. The model group exhibited impaired spatial recall testing, evidenced by reduced search strategy scores (1.82 ± 0.65 vs 2.63 ± 0.69 in the control group) despite comparable swim speeds. While the total target quadrant distance and the visit time showed no significant differences, the model animals demonstrated significantly lower target quadrant visit times and three ratios to total value, confirming long‐term memory deficits (Figure S14f–m, Supporting Information).

Following transplantation, all the groups demonstrated improved search pattern scores and reduced time and distance to reach the platform during training, indicating effective learning (Figure 6g,i; Figure S14n, Supporting Information). In the final test, the preserved swim speeds across the groups (Figure S14o, Supporting Information) confirmed motor function remained intact throughout interventions. The Ctrl group all exhibited trendy search mode, while the Exp group had two animals displaying random search mode. The Cell group showed a mix of random and peripheral search patterns, and the Mat group had two animals with peripheral search patterns, reflecting a gradient of memory capabilities (Figure 6h). Similar trends were observed in the target quadrant distance and the visit times. Significant differences were found between the Ctrl and the Mat groups, as well as between the Exp and the Mat groups. A similar trend was observed in the target quadrant visit duration, but the significance was not as high (Figure 6j). In terms of ratios to total values, except for visit times, other indicators showed trends but no significant differences (Figure S14p, Supporting Information). The discrepancy in results between the NOR and MWM tests may be attributed to the inherent complexity of animal experiments, differences in modeling and testing timelines, as well as the distinct cognitive characteristics assessed by each paradigm. Previous studies have also reported inconsistencies between NOR and MWM outcomes.^[^ ^72^, ^73^ ^]^ The NOR test primarily evaluates recognition memory, which is relatively less dependent on the hippocampus and represents a low‐stress, simple task. In contrast, the MWM test mainly assesses spatial memory, which is highly hippocampus‐dependent and constitutes a high‐stress, complex task. Such differences between the two behavioral paradigms may be related to the spatially restricted initial pathology within the hippocampus^[^ ^74^ ^]^ and its slow temporal progression.^[^ ^75^ ^]^ Overall, the water maze results clearly indicated that construct transplantation significantly improved long‐term learning and memory capabilities in model animals to levels comparable to healthy controls. Consistent with other studies, cell suspension transplantation also showed some effectiveness^[^ ^76^ ^]^ but was less effective compared to construct transplantation.

### 2.6 Constructs Transplantation Potentiated Hippocampal LTP Underlying Cognitive Enhancement

Long‐term potentiation (LTP) is a synaptic plasticity mechanism essential for learning and memory.^[^ ^77^ ^]^ LTP involves activity‐dependent strengthening of synaptic connections through increased AMPA receptor density and modifications of postsynaptic proteins like PSD‐95. Semi‐quantitative analysis of PSD‐95 expression in hippocampal slices demonstrated that construct transplantation significantly restored synaptic protein levels compared to the Mat and the Cell groups. This molecular restoration aligned with observed improvements in long‐term spatial memory, suggesting that the enhanced LTP function underlay the cognitive recovery.

**Figure 7:**
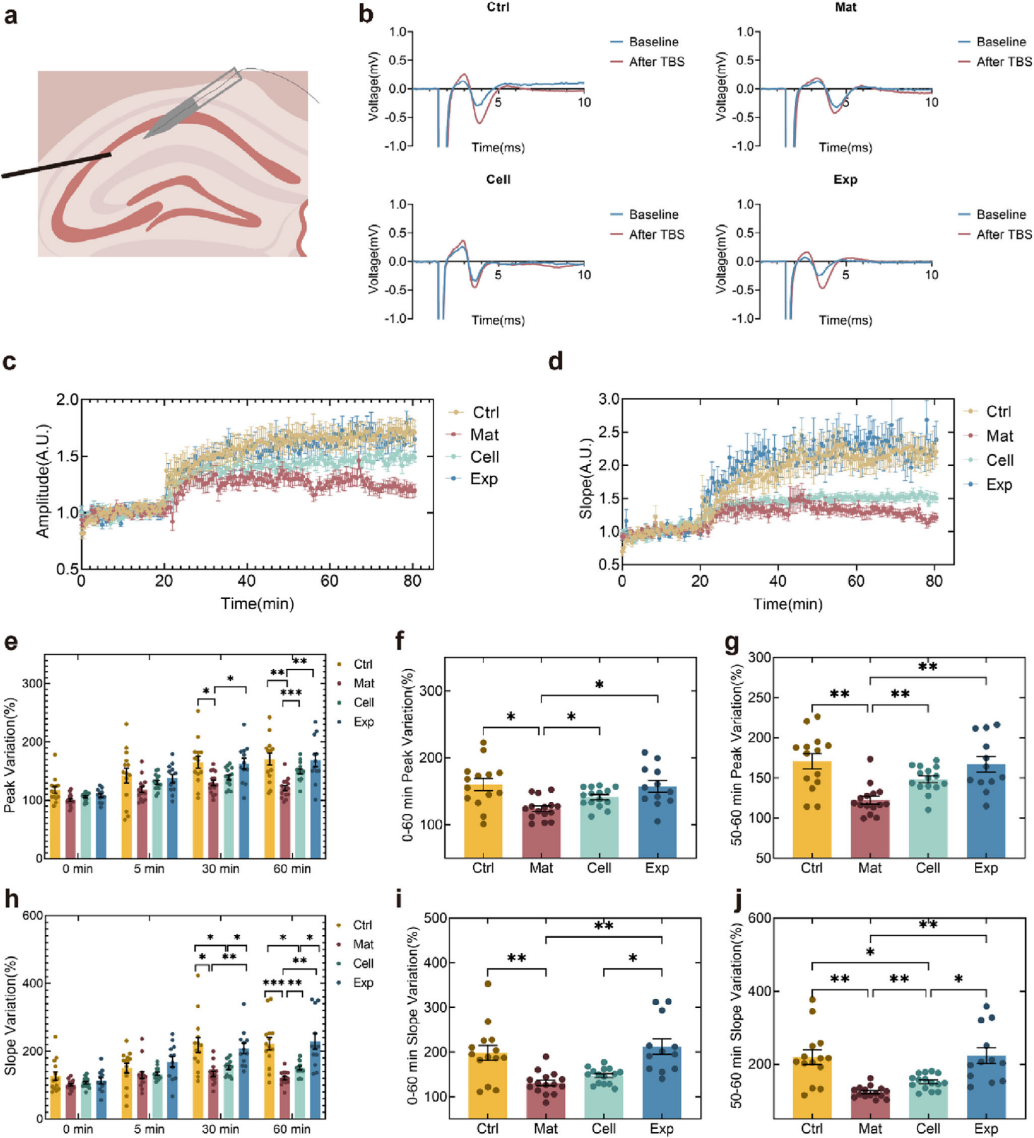
Functional restoration of hippocampal LTP through engineered construct transplantation. a) Experimental schematic for LTP recording. Synaptic stimulation was delivered to Schaffer collaterals via bipolar electrodes, with fEPSPs recorded from pyramidal layer of CA1 using glass microelectrodes. b) Representative fEPSP traces at baseline and after TBS in the Ctrl, Mat, Cell, and Exp groups. c,d) Normalized fEPSP amplitude and slope across groups. e) Normalized fEPSP amplitude at 0, 5, 30, and 60 min after TBS. The data were calculated using three consecutive data points centered around each time point. f) Mean normalized fEPSP amplitude during 0–60 min post‐TBS, reflecting the enhancement. g) Mean normalized fEPSP amplitude during 50–60 min post‐TBS, reflecting the maintenance. h–j) Normalized fEPSP slope, calculated in a manner consistent with (e–g). Data presented as mean ± SEM. *n* = 14, 15, 15, 12. For panels (e and h), Two‐way Welch's ANOVA with Games–Howell post hoc tests was applied. For panels (f, g, i), and (j), Welch's ANOVA with Dunnett's T3 multiple comparisons test was used. *p < 0.05, **p < 0.01, ***p < 0.001, and ****p < 0.0001.

LTP assessments were performed on acute hippocampal slices. Stimulation electrodes were positioned at Schaffer collaterals while recording electrodes monitored field excitatory postsynaptic potentials (fEPSPs) in the CA1 pyramidal layer (**Figure** **7**a). After a 20‐min baseline recording, theta‐burst stimulation (TBS) was applied, followed by a 60‐min monitoring. All groups exhibited immediate fEPSP amplitude and slope increase post‐TBS, confirming intact basal synaptic transmission. However, the Mat group showed rapid fEPSP decay, indicating impaired LTP maintenance. Both the Cell and the Exp groups demonstrated significant recovery, with Exp achieving near‐control‐level LTP persistence (Figure 7b,c).

A comparative analysis was conducted at key time points (0, 5, 30, and 60 min) to further quantify the dynamics of LTP. Group differences in both fEPSP amplitude and slope emerged shortly after TBS, following the hierarchy Ctrl > Exp > Cell > Mat. These differences became statistically significant at 30 min and remained sustained at 60 min. Based on these observations, the LTP outcomes were further evaluated from two perspectives: enhancement and maintenance. Enhancement consisted of the fEPSP from the end of TBS until approximately 1 h post‐induction (0–60 min). Maintenance consisted of the fEPSP recorded during the 50–60 min interval and was used as a quantitative measure, consistent with previously established methodologies.^[^ ^78^ ^]^ As shown in Figure 7d,e, from the perspective of overall enhancement the fEPSP amplitudes, which indicated signal transmission strength, were significantly higher in the Ctrl, the Exp, and the Cell groups compared to the Mat group. There were no significant differences among the Ctrl, the Exp, and the Cell groups. This demonstrated the successful establishment of the model and the effectiveness of stem cell transplantation. In terms of the overall fEPSP slope, which reflected signal transmission speed, the Exp group showed a significant difference from the Cell group. The difference highlighted the advantage of construct transplantation over cell suspension injection. This conclusion became even more pronounced in the maintenance phase (Figure 7f,g), suggesting that construct transplantation might play a more significant role in long‐term memory retention. Taking the fEPSP amplitude at 50–60 min as a reference, and using the Ctrl group as the baseline (100%), the LTP function in the Mat group decreased to 71.69% ± 10.91%, while it recovered to 97.89% ± 19.84% in the Exp group and reached 86.99% ± 9.60% in the Cell group. The typical fEPSP changes for each group, as displayed in Figure 7h–k, also reflected the trend of Exp>Ctrl>Cell>Mat in terms of the degree of change.

The therapeutic constructs likely facilitated LTP by stabilizing synaptic architectures, as evidenced by preserved SYP and PSD‐95 levels in transplanted animals. While the cell suspension transplants showed partial synaptic protein recovery, their irregular spatial distribution correlated with weaker behavioral outcomes. In light of the enhanced cell retention (3.41‐fold increase) and improved LTP recovery (97.89% of healthy control levels) following construct transplantation, it was proposed that the transplant promoted neuronal survival and integration, leading to functional repair of hippocampal circuitry. This recovery may underlie the observed search strategy shift from peripheral search pattern to a more efficient trendy search mode, suggesting restored advanced spatial memory function.

## 3 Conclusion

This study developed a TTBT(3‐component 3D Brain‐like Transplant) system for Alzheimer's disease transplantation therapy. The constructed 3D‐printed neural stem cell graft demonstrated promising therapeutic effects in an Alzheimer's‐like dementia animal model. Compared with cell suspension injection, the graft showed potential advantages in ameliorating neural network integrity, tau pathology, neuroinflammation, cognitive behavior, and LTP function.

These therapeutic effects are derived from the design strategy tailored to AD‐specific requirements, integrating three critical aspects: NPCs serving as renewable progenitor alternatives, hydrogel material mimicking native brain mechanics (1–4 kPa compressive elastic modulus), and dot‐shaped constructs (1 mm diameter) engineered for minimally invasive hippocampal transplantation. The 3D‐printed constructs demonstrated 3.41‐fold enhanced cellular retention over the cell suspension group. Microenvironmental control increased neuron differentiation to 79.21 ± 6.67% (NeuN) compared to 65.08 ± 7.14% in suspensions, with GABAergic subtyping nearly doubling (29.85 ± 7.69% vs 15.93 ± 10.33% GAD65). Crucially, structural confinement suppressed astrocyte differentiation (21.18 ± 4.50% vs 37.62 ± 21.25% GFAP), decreasing possible reactive astrocytes post‐transplantation and thereby potentially reducing the harm caused by the transplantation itself and improving the overall therapeutic effect. Benefiting from enhanced cell retention and improved control over differentiation direction, transplantation of the graft led to improved neural network repair, reduced neuroinflammatory responses (as indicated by lower levels of TNF‐α, IL‐1β, IL‐6, and GFAP), and amelioration of tau‐related pathology compared to cell suspension transplantation. These improvements were ultimately reflected in superior search strategies and enhanced spatial learning and memory performance in the Morris water maze, along with a corresponding recovery of long‐term potentiation (LTP) (Exp: 97.89% ± 19.84% vs Cell: 86.99% ± 9.60%). This study provides initial evidence that a 3D‐bioprinted NPC transplant mitigated multiple aspects of tauopathy‐related deficits in an acute neurotoxic AD‐like model, suggesting a potential strategy for future therapeutic development.

The translational implications of this work were constrained by limitations in modeling chronic neurodegeneration. The young animal model, while developing tau‐driven cognitive deficits, failed to exhibit Aβ plaque pathology characteristic of advanced AD. The 1‐month observation window, though sufficient to capture acute therapeutic effects, was not capable of predicting outcomes in the decades‐long neurodegenerative course. Besides, the current 1‐mm construct diameter reflected an optimization balancing therapeutic effects and surgical feasibility in animal models rather than the technical limitations of 3D bioprinting itself. For human treatment, constructs of larger size have been proven workable in vitro but still need to be verified in big animals in vivo. Furthermore, the mechanisms underlying graft‐host circuit integration remained unexplored. Future studies employing in vivo two‐photon calcium monitoring, optogenetic mapping, and tissue‐clearing techniques would elucidate these critical neurorestorative processes.

Despite these challenges, this work provided foundational evidence that engineered NPC constructs could achieve multiscale repair from the molecular level to behavioral performance. The optimized materials successfully improved cell repairments in the AD‐like model. By bridging developmental biology with precision biomanufacturing, a potential new therapeutic framework has been established that could extend to other brain diseases with similar defects, such as traumatic brain injury (TBI). In the future, comprehensive investigations into in vivo graft‐host connectivity and circuit recovery will be conducted in collaboration with neuroscience experts. Additionally, the development of injectable biomaterial systems that can preserve structural integrity post‐transplantation while maintaining neural biocompatibility is also crucial for large animal experiments.

## 4 Experimental Section

### NPC Culture Protocol

HiPSCs (DYR‐0100, ATCC, USA) were cultured in mTeSR Plus medium (100‐0276, STEMCELL Technologies, USA) until ready for passage. Cells were dissociated from the culture plate into single cells using Gentle Cell Dissociation Reagent (100‐0485, STEMCELL Technologies, USA). These single iPSCs were then suspended in STEMdiff Neural Induction Medium (08581, STEMCELL Technologies, USA) containing 10 µm Y‐27632 (72302, STEMCELL Technologies, USA) and plated onto Matrigel (354277, Corning, USA)‐coated plates. The medium was completely replaced with STEMdiff Neural Induction Medium daily until the culture was ready for passage. Cells were passaged twice using ACCUTASE (07920, STEMCELL Technologies, USA). These NPCs were maintained in a 37 °C incubator with 5% CO_2_, and the STEMdiff Neural Progenitor Medium (05833, STEMCELL Technologies, USA) was changed daily. All cells used in this study were within five passages. Cells were labeled with mCherry fluorescent protein via lentiviral transfection (BrainVTA, China) and subsequently screened for one generation using puromycin (ST551, Beyotime, China) before use.

### Bioink Preparation

The bioink comprised three components: gelatin, sodium alginate, and fibrinogen. A 10% (w/v) and 20% (w/v) gelatin(V900863, Sigma, USA) stock solution was prepared using 0.9% (w/v) sodium chloride, pasteurized, and stored at 4 °C. Sodium alginate (A2033, Sigma, USA) was prepared as a 1% (w/v) stock solution, sterilized by pasteurization, and stored at 4 °C. Fibrinogen (S12024, Source Leaf, China) was dissolved in 0.9% (w/v) sodium chloride to a concentration of 4% (w/v) and stored at −20 °C. The bioink was formulated by mixing these three components with the cell suspension in a ratio of 2:1:1:3, resulting in final concentrations of 5.71% or 2.86% for gelatin, 0.14% for sodium alginate, 0.57% for fibrinogen, and a cell concentration exceeding 6.67 × 10⁶ cells·mL^−1^.

### Material Testing—Elastic Modulus Testing

The elastic modulus of the crosslinked bio‐ink was tested using the Bose ElectroForce 3200 compression system (Bose Corporation, USA). Samples with a diameter of 6 mm and a height of 4 mm were formed through nylon molds and crosslinked in situ. After culturing for 1, 4, 7, 10, and 14 days, the samples were subjected to compression elastic modulus analysis. During the test, the samples were compressed to 75% of their original height at a speed of 1 mm/min, with force and displacement recorded. The elastic modulus was calculated through linear fitting of the stress–strain data between strains of 0 to 0.1.

### Material Testing—Rheological Property Testing

The rheological properties of the bioink were measured using a rheometer (MCR301, Anton Paar, Austria) with a 25 mm diameter plane plate. The tests were conducted at 1% strain and a sample frequency of 1 Hz, with amplitude and frequency scans adjusted to ranges of 0.1–100% and 0.1–10 Hz, respectively. In the temperature scan, the temperature was gradually reduced from 37 to 4 °C. For shear thinning, shear modulus testing, amplitude, and frequency scans, temperatures of 4, 37 °C, and the crosslinking point temperature were selected.

### Material Testing—FTIR Analysis

FTIR analysis was performed on DAYs 1, 7, and 14 using pure gelatin, sodium alginate, and fibrinogen as controls to evaluate the stability of the bio‐ink components. The samples were rapidly cooled in liquid nitrogen and then freeze‐dried using a freeze dryer (LGJ‐12, Songyuan Huaxing Technology, China), followed by analysis on a Nicolet iS50 Fourier Transform Infrared Spectrometer (Thermo Fisher, USA).

### Material Testing—Microscopic Morphology Observation

The same method was used to prepare samples for microscopic morphology observation, which were then gold‐sprayed and imaged on a GeminiSEM 300 SEM (Zeiss, Germany).

### Material Testing—Shape Fidelity and Degradation Testing

Shape fidelity and degradation tests were conducted on structures of the same shape with and without cells, maintaining consistent printing and crosslinking procedures. The samples were cultured in artificial cerebrospinal fluid (aCSF) containing penicillin and streptomycin and collected at specified time points. The dimensions and weight were measured using a stereomicroscope (SMZ800N, Nikon, Japan) and a PX124ZH electronic balance (Ohaus, China). These comprehensive tests provided important data on the mechanical properties, rheological behavior, component stability, microstructure, shape fidelity, and degradation characteristics of the bio‐ink, supporting its further research and application development.

### Construct Printing and Subsequent Culturing Process

For the targeted in vivo transplantation site and size, a dot construct with dimensions of Φ1 mm × 0.25 mm was designed. The curvatures were calculated by the Kappa plugin from Fiji. A grid structure with dimensions of 7 mm × 7 mm × 0.75 mm, a line spacing of 1.2 mm, and a single‐layer height of 0.25 mm was designed to validate the potential of the bioink for constructing customized transplants. The bio‐printer (BIOMAKER 4, SUNP BIOTECH, China) was disinfected with ultraviolet light for 20 min before use to ensure sterility. Through process optimization, the printing parameters for the final construct were adjusted to: nozzle temperature of 13 °C, base temperature of 10 °C, needle inner diameter of 0.30 mm, printing speed of 5 mm·s^−1^, and extrusion speed of 1 mm^3^·s^−1^.

After preparation, the bioink was loaded into a 1 mL syringe and pre‐cooled at 4 °C for 4 min before printing. The syringe was then installed on the printer, and the printing process was automatically executed using the predetermined parameters. Immediately after printing, the structure was transferred to an ice plate and chemically crosslinked with 2% CaCl_2_ for 3 min. Subsequently, the culture medium containing 10 U·mL^−1^ thrombin (S10117, Yuanye, China), 0.36 U·mL^−1^ transglutaminase (S10156, Yuanye, China), and 100 U·mL^−1^ penicillin 0.1 mg·mL^−1^ streptomycin (C0222, Beyotime, China) was added, and the structure was incubated at 37 °C for 2–4 h. For continuous culturing, STEMdiff Neural Progenitor Medium was supplemented with 20 µg·mL^−1^ aprotinin (S10089, Yuanye, China) and penicillin–streptomycin solution. After one week, the medium was switched to Neurobasal Medium (Gibco, USA) with the same supplements for self‐differentiation function testing.

### In Vitro Cell Test—Cell Viability

Cell viability was detected using Calcein AM and PI (C542, DOJINDO, Japan), applied to the structures at concentrations of 2 µM and 4.5 µM, respectively. A laser scanning confocal microscope (FV3000, Olympus, Japan) was used to excite green (live cells) and red (dead cells) fluorescence with 488 nm and 561 nm lasers, respectively, to acquire Z‐series images. Cell counting was performed using randomly selected images and the “Cell Counter” tool in Fiji open‐source software.

### In Vitro Cell Test—Transcriptome Analyses

RNA sequencing samples were obtained by hydrolyzing the structures. The structures were digested in PBS containing 0.5 mg·mL^−1^ collagenase I (ST2294, Source Leaf, China) and calcium ions for 1 h to obtain 3D cell groups, while 2D cells were directly dissociated from the culture dishes using ACCUTASE. The cells were washed three times with PBS, and a working lysis buffer containing RNAase inhibitor (PLASTECH, China) was added before storing at −80 °C. The samples were sent to the PLASTECH company, and libraries were constructed according to the DRUG‐seq2 protocol, followed by sequencing using the Illumina Novaseq platform. Gene enrichment results were analyzed using DAVID,^[^ ^79^ ^]^ and expression quantity results were analyzed for quality and differences using R language and corresponding packages. All result graphs were plotted using R language and online websites.^[^ ^80^, ^81^ ^]^

### In Vitro Cell Test—IF

The IF was used to detect GFAP (50‐9892‐82, Thermo, USA), Nestin (60091AD, STEMCELL Technologies, USA), NeuN (R010222, Millipore, USA), and TUBB3 (53‐4510‐82, eBioscience, USA). Samples were collected on DAYs 7 and 14, washed twice with DPBS, and fixed with 4% paraformaldehyde for 30 min. After three washes, the samples were treated with DPBS containing 0.1% Triton‐X 100 and 0.1% bovine serum albumin (BSA) for 30 min to increase permeability. Subsequently, the samples were blocked in DPBS containing 2% BSA for 1 h. Then, the samples were incubated overnight at 4 °C in DPBS containing 0.1% BSA and diluted primary antibodies (with DAPI, Invitrogen, USA). For antibodies requiring secondary antibodies, the samples were further incubated in DPBS containing 0.1% BSA, the corresponding secondary antibody, and DAPI, in the dark for 1 h after three washes. After three final washes, the samples were ready for imaging. A laser scanning confocal microscope (FV3000, Olympus, Japan) was used to excite fluorescence labels with 640 nm, 594 nm, 488 nm, and 405 nm lasers.

### In Vitro Cell Test—Calcium Signaling

Calcium ion detection was performed using Fluo 4‐AM (F311, DOJINDO, Japan) to assess the activity of live cells or neural function within the constructs. After washing the constructs three times with HBSS, they were incubated with 5 µM Calcein AM at 37 °C for 45 min. After another three washes with HBSS, the constructs were observed using a laser confocal microscope (FV3000, Olympus, Japan) with 488 nm laser excitation, with each recording lasting 10 min. Cell recognition was assisted by CellPose,^[^ ^82^ ^]^ and signal analysis was performed using MATLAB.

### Animal Transplantation Experiment

Male Wistar rats aged 6 weeks, weighing approximately 200 g, were selected for the experiment and acclimatized for 5 days before the start of the experiment. The animals were divided into a model group and a control group. Rats in the model group were given oral gavage of AlCl_3_ at 500 mg/kg daily for 30 consecutive days to induce an AD‐like dementia model. The control group received oral gavage of saline and served as healthy controls.

NPC constructs were transplanted on the DAY 5 post‐printing. The constructs were aspirated using a 1 mL syringe with negative pressure for injection, with approximately 2300 cells per construct. The rats were anesthetized with isoflurane, and after applying erythromycin to the eyes, they were fixed in a stereotaxic apparatus. The local area was shaved and disinfected with iodine, and the scalp was incised. A hole was drilled in the skull at the corresponding location using a drill. An incision was made to the cerebral cortex using a syringe, and then the syringe containing the construct was used to transplant it to the lateral CA1 region of the bilateral hippocampus, with one construct per side. The wound was sutured postoperatively. For the NPC suspension transplantation, the cells were collected and resuspended in saline. A 1 mL syringe was used to aspirate a total of 2300 cells in a volume equal to that of the construct (0.350 µL), and the transplantation location and number were the same as those in the construct group. For the empty material group, cell‐free constructs of the same volume were prepared and underwent the same transplantation procedure. For the control group, an equal volume of normal saline was injected.

The animal experiment was approved by the Animal Welfare Ethics Committee of Beijing MDKN Biotechnology Co., LTD., and was conducted in strict accordance with the experimental animal care and use guidelines of the Beijing Animal Control Committee. (Approval No. MDKN‐2024‐032).

### Brain Slice IF and IHC

Antibodies used: NeuN (R010222, epizyme, China), GFAP (50‐9892‐82, Thermo, USA), TUBB3 (53‐4510‐82, eBioscience, USA), SYP (82900‐1‐RR, Proteintech, China), PSD‐95 (81106‐1‐RR, Proteintech, China), tau (66499‐1‐lg, Proteintech, China), P‐tau (28866‐1‐AP, Proteintech, China), Aβ (Ab201060, abcam, UK).

For IF staining, brain slices were fixed with paraformaldehyde for 24‐48 h, followed by three washes with PBS, each for 5 min. The slices were then blocked with blocking solution (2% BSA, 0.3% Triton‐X 100) at room temperature for 2 h. After three more washes with PBS, primary antibodies were incubated overnight at 4 °C. The slices were then washed three times with PBST, each for 5 min, followed by incubation with secondary antibodies at room temperature for 2 h. After another three washes with PBST, the slices were observed under a laser confocal microscope (FV3000, Olympus, Japan).

For IHC staining, brain slices were fixed with paraformaldehyde for 24–48 h, followed by gradient dehydration, clearing, and wax immersion. The tissue sections were embedded with the cut surface facing down, cooled, and then removed. The wax blocks were pre‐cooled for 20 min before sectioning into 4 µm thick slices using a paraffin microtome (RM2235, Leica, Germany). The slices were spread in 42 °C water, mounted, and baked in a 65 °C oven for 1 h. Antigen retrieval was performed by boiling the slices in Tris‐EDTA in a pressure cooker for 3 min. The slices were then washed three times with PBS, each for 5 min, followed by incubation with 3% H_2_O_2_ at room temperature for 20 min to block endogenous enzymes. After another three washes with PBS, the slices were blocked with 10% goat serum at 37 °C for 30 min. Primary antibodies were incubated overnight at 4 °C, followed by three washes with PBST, each for 5 min. Secondary antibodies were incubated at room temperature for 1 h, followed by three more washes with PBST. DAB working solution was applied for color development. The slices were then stained with hematoxylin for 3‐5 min, rinsed with water, differentiated in 0.5% hydrochloric acid alcohol for 1–2 s, and soaked in bluing solution for 3–5 s before being rinsed with water again. The tissue was then dehydrated, cleared, mounted and observed using a slide scanner (Pannoramic SCAN, 3DHISTECH, Hungary).

All fluorescence images used for statistical analysis underwent the same staining process and imaging conditions, with DAPI as the standard channel for ratio correction to obtain corrected fluorescence intensity for semi‐quantitative analysis.

### Animal Behavioral Experiments

Animal behavioral experiments were conducted at two time points: after the completion of the AD‐like model and one month after transplantation. The experiments included the open field test, novel object recognition test, and water maze test, performed in sequence.

### Animal Behavioral Experiments—Open Field Test

For the open field test, spanning two days with one day for adaptation and another for formal testing, animals were placed in an empty testing box to record their movement trajectories for 5 min.

### Animal Behavioral Experiments—Novel Object Recognition Test

For the open field test, spanning two days with one day for adaptation and another for formal testing, animals were placed in an empty testing box to record their movement trajectories for 5 min. The novel object recognition test, also over two days, involved a training day where animals explored two objects in a box for 300 s, followed by a testing day with one object replaced by a new one, again recording trajectories for 300 s.

### Animal Behavioral Experiments—Water Maze Test

The water maze test lasted six days, with a 5‐day learning phase where rats, starting from different quadrants, searched for a hidden platform in the first quadrant, and a 1‐day testing phase without the platform, both recording 60 s of movement trajectories.

### LTP Detection

Rats were anesthetized with isoflurane and then perfused with saline in vivo. Fresh brain tissue was extracted, and coronal slices, including the hippocampus and surrounding areas, with a thickness of 300 µm were cut using a vibratome (DTK‐1000N, DOSAKA, Japan) operating at 100 Hz and a speed of 0.5 mm/s. The slices were then incubated in oxygenated aCSF at 33.5 °C for 30 min. LTP in the brain slices was measured using a patch‐clamp system (MultiClamp 700B, Molecular Devices, America). Bipolar electrodes served as the stimulating electrodes, and glass electrodes filled with aCSF were used to record the signals. Oxygen was continuously supplied to the perfusion solution (aCSF) during the measurement. Stimulation was performed near the Schaffer collaterals in the CA3 region, and signals were read in the CA1 region. Initially, pulse stimulation was used to determine the io curve of the brain slice, and the current that elicited half the maximum voltage was selected for stimulation. Electrical stimulation was administered every 30 s, and the signal was recorded for 20 min as a baseline. Theta burst stimulation (TBS) was then applied with high‐frequency stimulation at 100 Hz for 0.1 s, repeated four times. After the stimulation, once the bubbles dissipated, electrical stimulation was administered every 30 s, and the signal was recorded for 60 min.

### Data Processing Methods

Unless otherwise specified, all data presented in the figures and text are expressed as mean ± standard error of the mean (SEM). Statistical analyses were performed using GraphPad Prism 9.5 and R software (version 4.4.3). A sample size of *n* ≥ 3 was used for all analyses, where statistical significance was assessed. For comparisons between two groups, the Mann–Whitney U test was applied to non‐normally distributed data. Normally distributed data were assessed for homogeneity of variances: Student's *t*‐test was used for data with equal variances, while Welch's *t*‐test was applied when variances were unequal. For comparisons among three or more groups, non‐normally distributed data were analyzed using the Kruskal–Wallis test followed by Dunn's multiple comparisons test. Normally distributed data were evaluated for homogeneity of variances: one‐way ANOVA with Tukey's post hoc test was used for data with equal variances, whereas Welch's ANOVA with Dunnett's T3 post hoc test was applied for data with unequal variances. For datasets involving two independent variables, non‐normally distributed data were analyzed using the Scheirer–Ray–Hare test, followed where appropriate by Dunn's multiple comparisons. Normally distributed data with equal variances were analyzed using two‐way ANOVA with Tukey's or Dunnett's T3 post hoc test, where significant effects were detected. For data with unequal variances, two‐way Welch's ANOVA was performed, followed by Games–Howell post hoc testing when significant differences were identified. The specific statistical tests used, their underlying assumptions, and sample sizes for each figure are summarized in Table S1 (Supporting Information). A corrected *p*‐value of less than 0.05 was considered statistically significant for all analyses. Significance levels are denoted as follows: *p < 0.05, **p < 0.01, ***p < 0.001, and ****p < 0.0001.

## Conflict of Interest

The authors declare no conflict of interest.

## Supporting information



Supporting Information

Supplemental Table 1

Supplemental DataFile

## Data Availability

All data supporting the findings of this study are available within the article and its supplementary materials. Requests for further information can be addressed to and will be provided by the corresponding authors.
