# Supplementary material for: Advanced Stem Cell Therapy: 3D‐Bioprinted Brain‐Like Transplants for Alzheimer's Disease‐Like Dementia
Source: Adv Sci (Weinh). 2025 Nov 7;13(4):e10062. doi: 10.1002/advs.202510062 (PMC12822481; doi:10.1002/advs.202510062)
Supplement: Supplementary file 1 — Supporting Information [file ADVS-13-e10062-s003.docx]

Supporting Information

# Advanced Stem Cell Therapy: 3D-Bioprinted Brain-like Transplants for Alzheimer’s Disease-like Dementia

*Ke Gai^1^, Dawen Gao^2^, Qingning Nie^2^, Xiao Luo^1^, Caizhe Xu^1^, Changhao Cai^1^, Austin Smith^1^, Xiang Li^3^, Wei Shi^2^, Lei Zhang^1^, Wei Sun^1^, Feng Lin^1*^, and Yu Song^1*^*

**Code S1. GCODE for All Constructs**

(separate file)


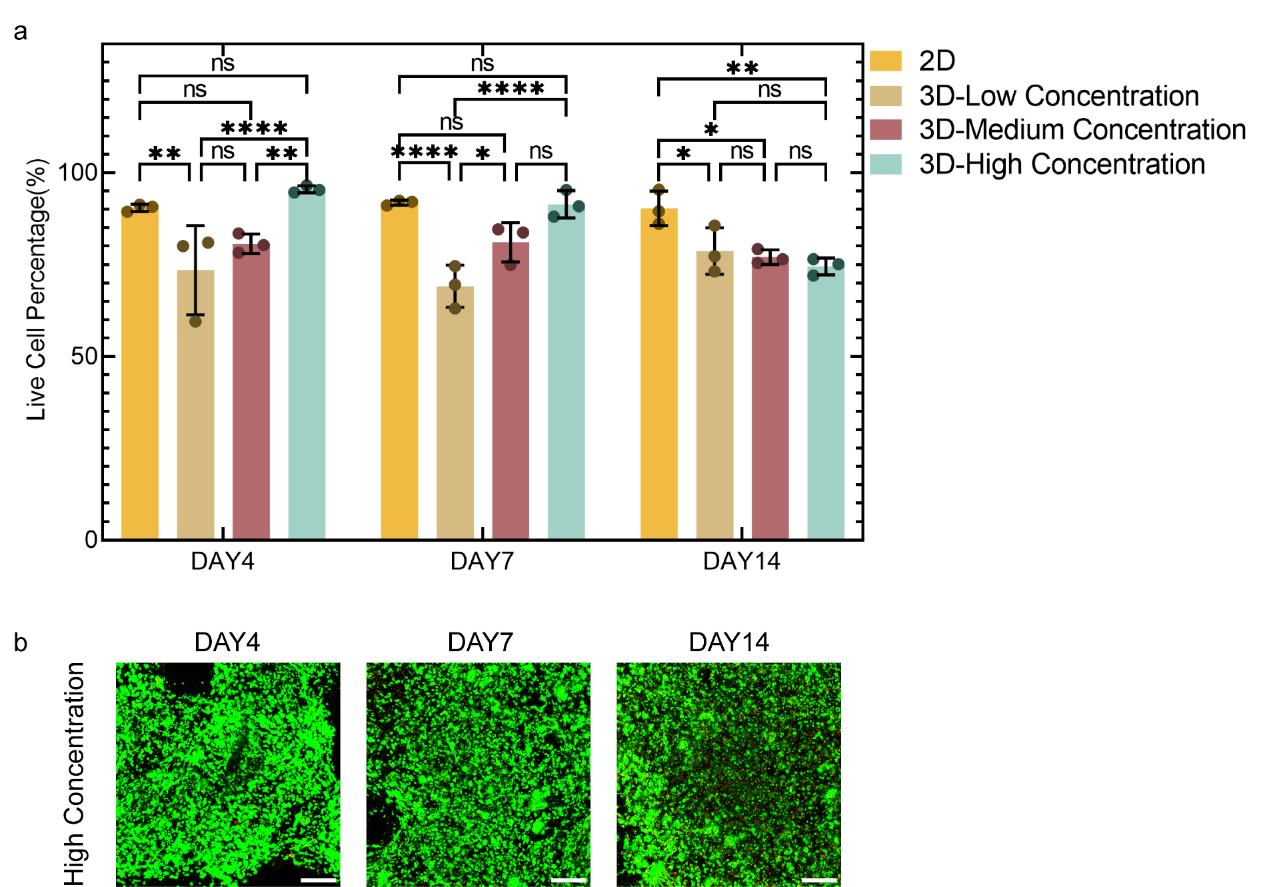


**Figure S1. Cell viability in bio-printed constructs at different cell concentrations.** **a**, Data presented as mean ± SD. (n = 3) P-values were calculated using two-way ANOVA followed by Tukey’s multiple comparisons test. *p < 0.05, **p < 0.01, ***p < 0.001, ****p < 0.0001. **b**, Cell status with high concentrations. Scale bars: 200 μm.


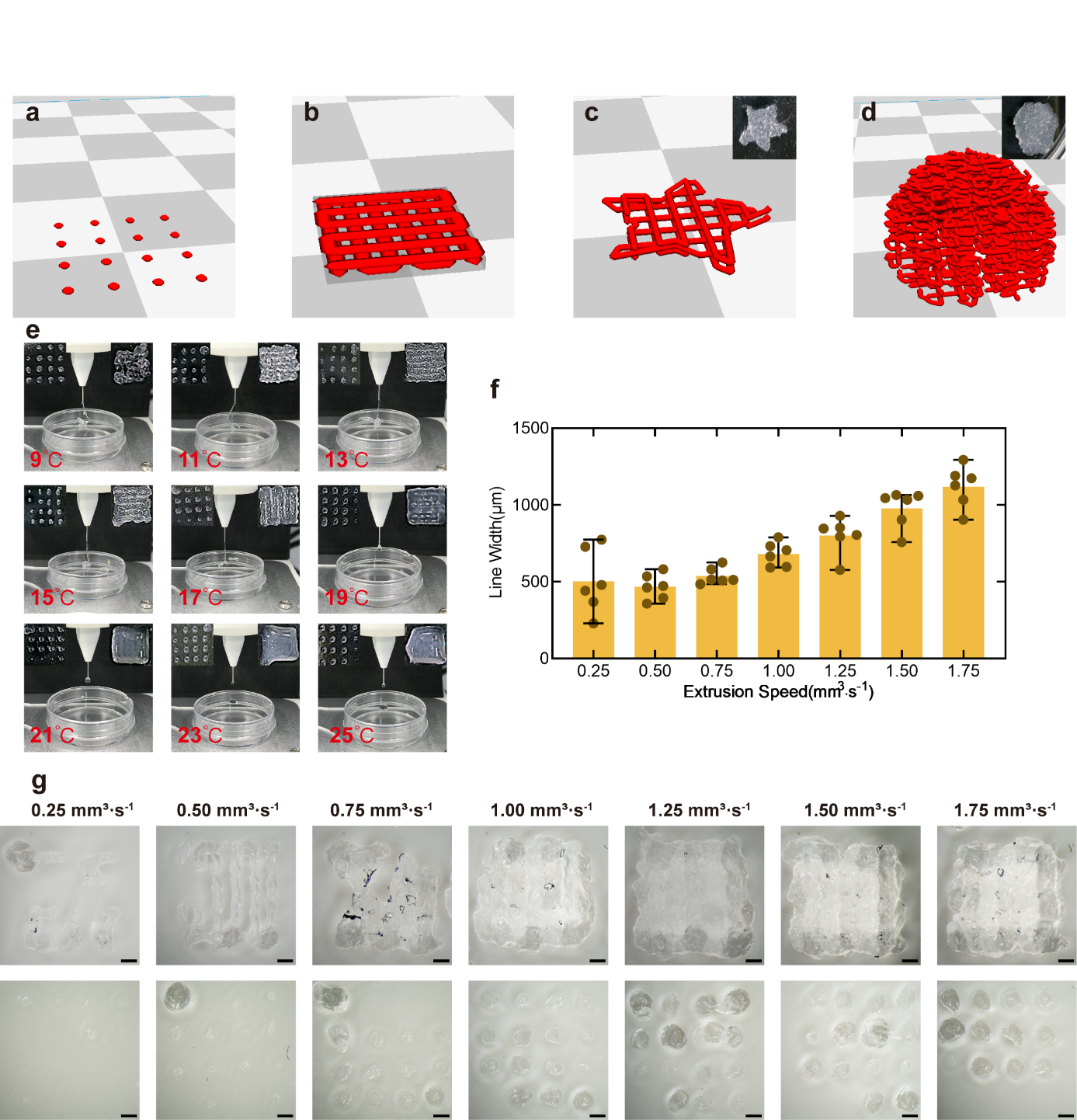


**Figure S1. Design and fabrication of 3D printed constructs. a-d**, Schematic illustrations of four distinct construct designs (dot, grid, star, and brain-mimetic architecture) with corresponding images of printed constructs for **(c,d**). Scale bars: 1 mm **e**, Temperature-dependent extrusion behavior and structural fidelity of bioink (9-25°C), demonstrating optimal printability between 11-15°C. **f**, Effects of extrusion speed on printed line width, presented as mean ± SD. (n = 6). **g,** The influence of extrusion speed on the size and forming effect of grid and dot structures (0.25-1.75 mm^3^·s^-1^). Scale bars: 1000 μm.


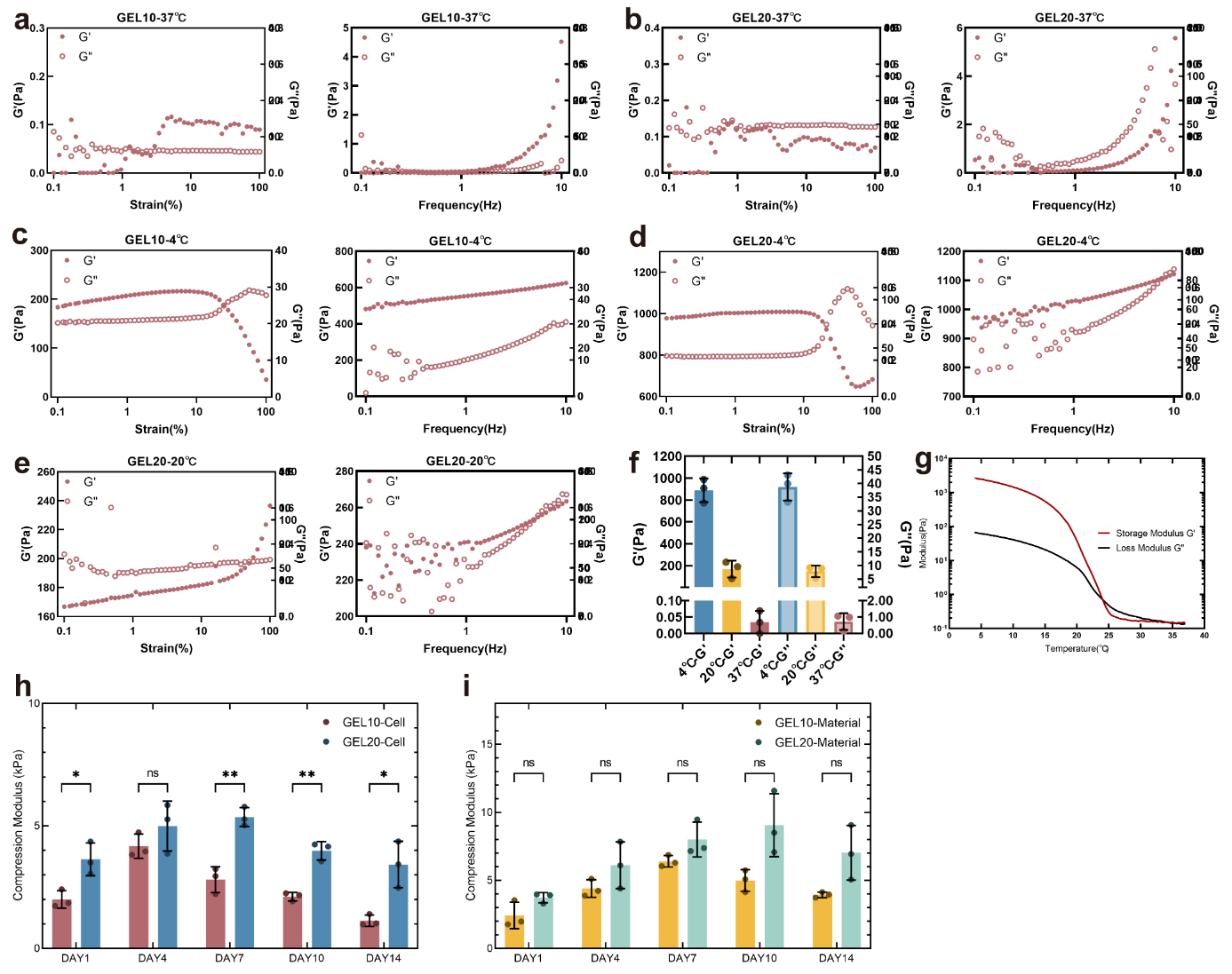


**Figure S3. Mechanical characterization and optimization of hydrogel systems.** **a-d,** Amplitude and frequency sweeps of GEL10 and GEL20 formulations at 37°C and 4°C temperatures. **e,** Amplitude and frequency sweeps of GEL20 at its gelation point (20 °C). **f,** Temperature-dependent shear modulus variations in GEL20. (n=3) **g,** Temperature sweep test showing storage (G') and loss (G'') moduli crossover. **h,i,** Time-dependent compressive modulus evolution of acellular and cell-laden constructs over 14-day culture. Data presented as mean ± SD. (n=3) Statistical comparisons performed using multiple unpaired t-tests.


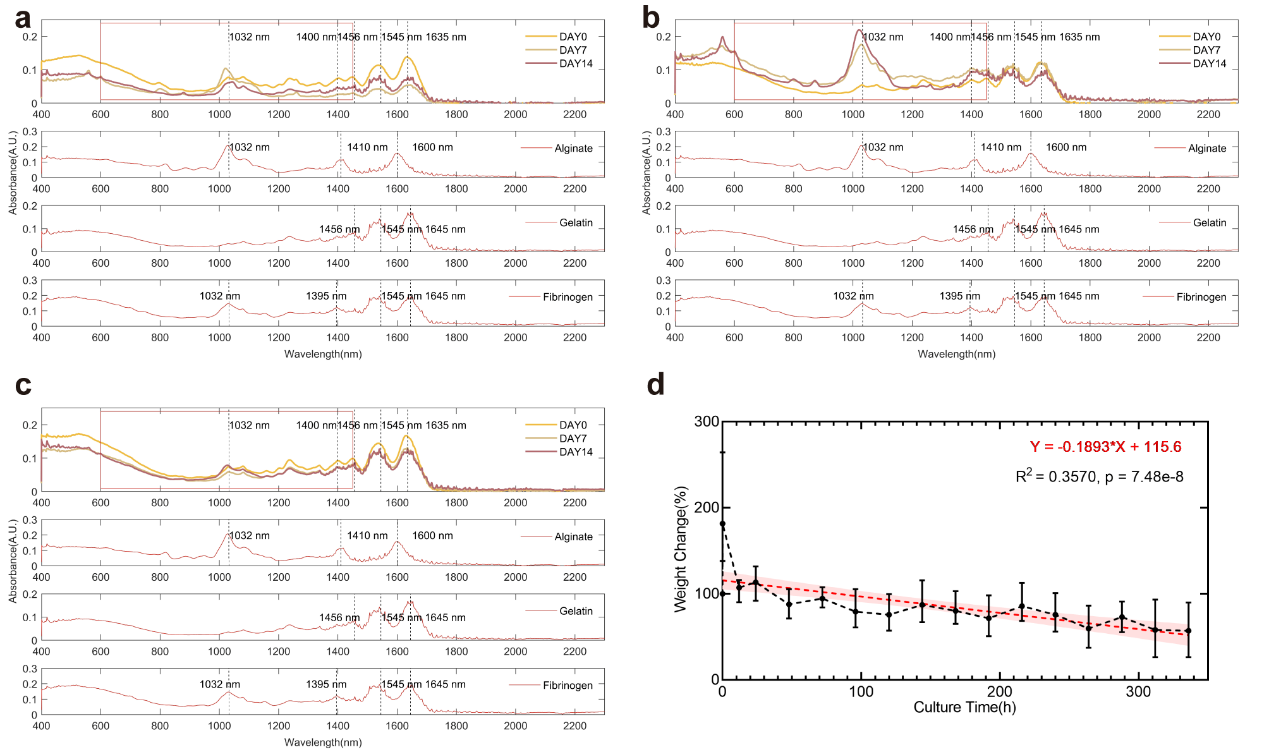


**Figure S3. Degradation of bioinks.** **a,** FTIR spectral analysis of acellular GEL10 constructs after 14-day physiological degradation, compared with pristine polymer components(alginate-gelatin-fibrinogen). **b,** FTIR spectral analysis of cell-laden GEL20. **c,** FTIR spectral analysis of acellular GEL20. (representative of n = 3). **d,** Degradation curve fitting of GEL10 system containing cells cultured for two weeks. Data presented as mean ± SD (n = 4).


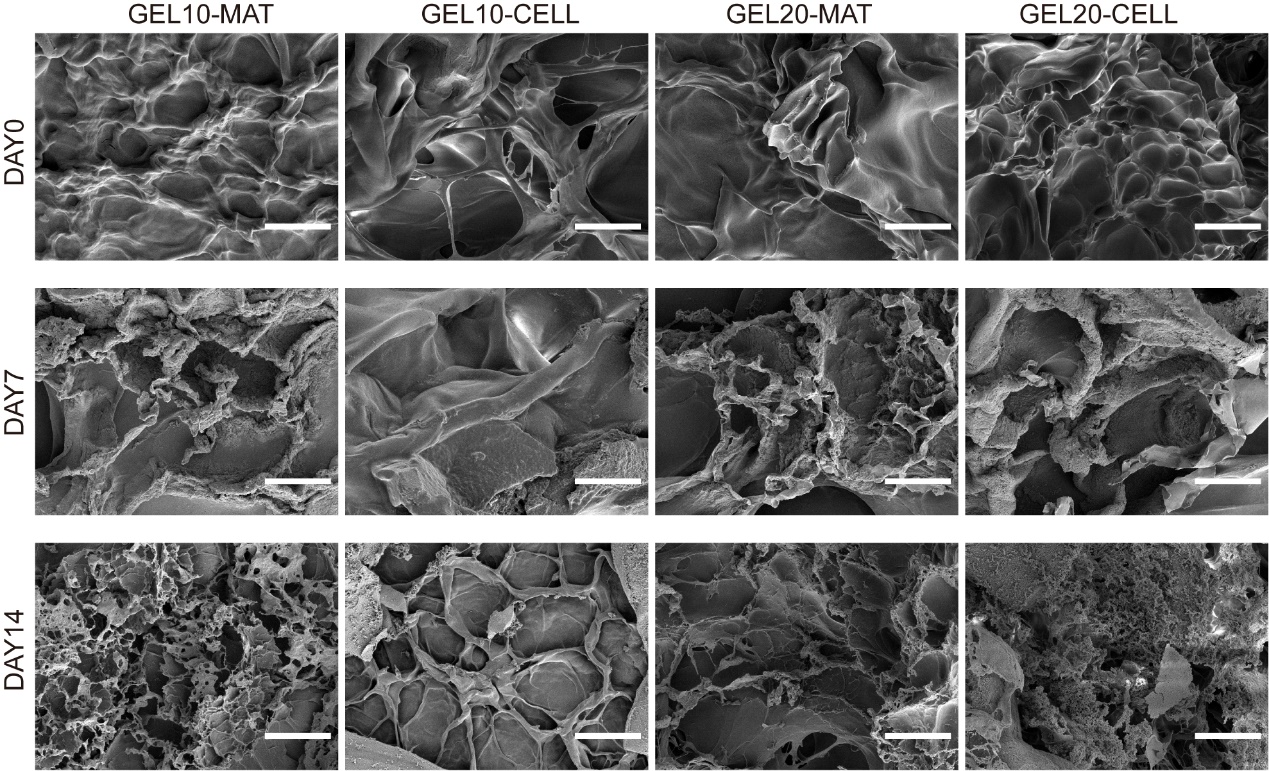


**Figure S4. Microstructure of cell-laden and acellular bioinks in 14-day culture.** Scale bars: 40 μm.


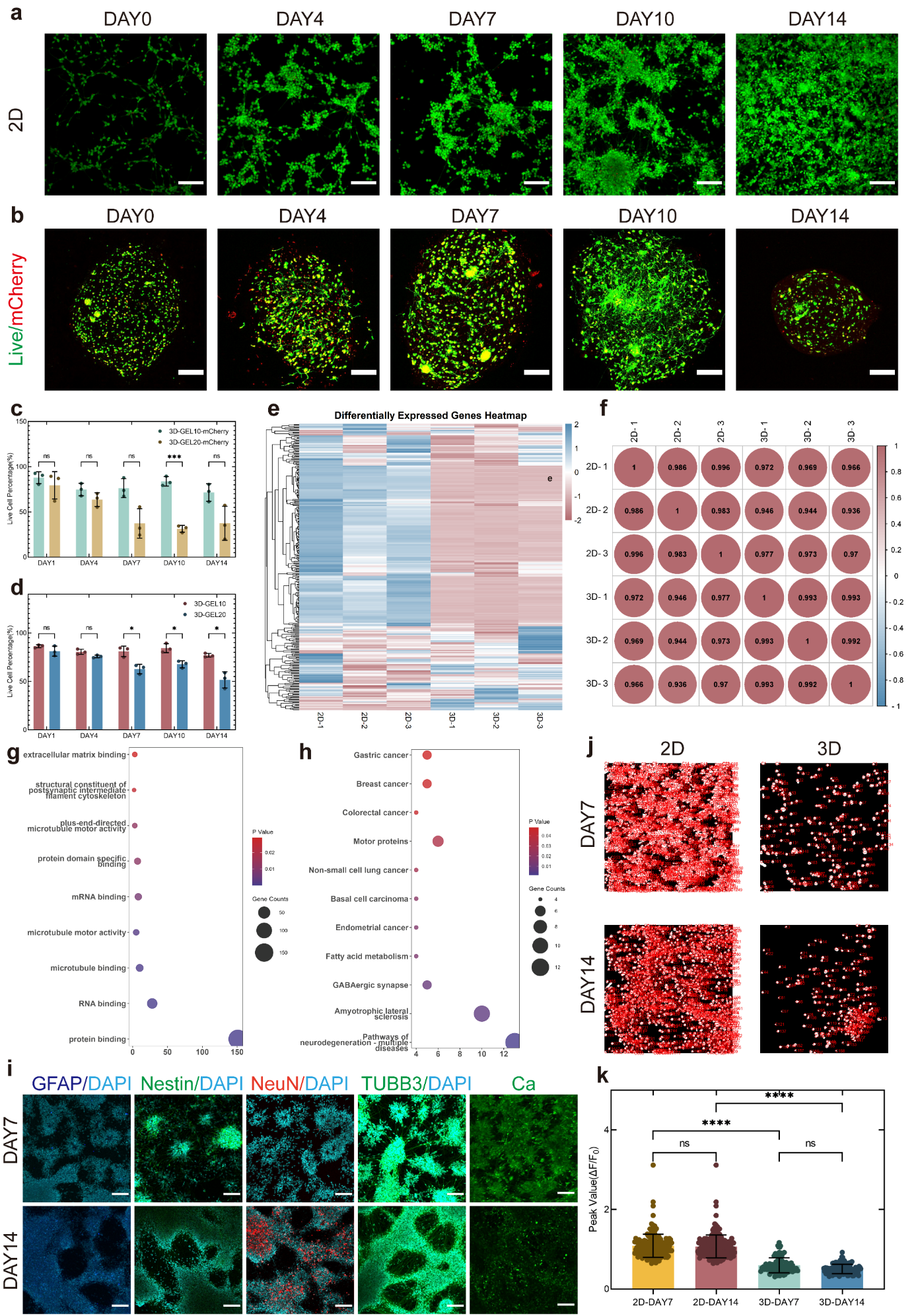


**Figure S6. Comparative analysis of cellular behavior in 3D constructs versus conventional 2D culture systems.** **a,b,**Viability assessment of 2D culture (Scale bars: 100 μm) and mCherry-labeled 3D constructs ( Scale bars: 200 μm) in the 14-day culture. Scale bars: 100 μm. **c,d,** Material-dependent NPC viability in GEL10 vs. GEL20 systems. Data analyzed with Multiple unpaired t tests. (n = 3) **e,f,** Heatmap of differentially expressed genes and correlation matrix. **g,h,** Enrichment in GO molecular function terms and KEGG pathways from DAVID. **i,** IF staining (GFAP/Nestin/NeuN/TUBB3) and Fluo-4 AM calcium imaging on days 7 and 14. Scale bars: 100 μm. **j,k,** ROI recognized in calcium imaging and peak value analysis. Data analyzed with Kruskal-Wallis test with Dunn's post-hoc comparisons. (n = 169, 187, 88, 116). Data presented as mean ± SD. *p < 0.05, **p < 0.01, ***p < 0.001, and ****p < 0.0001.


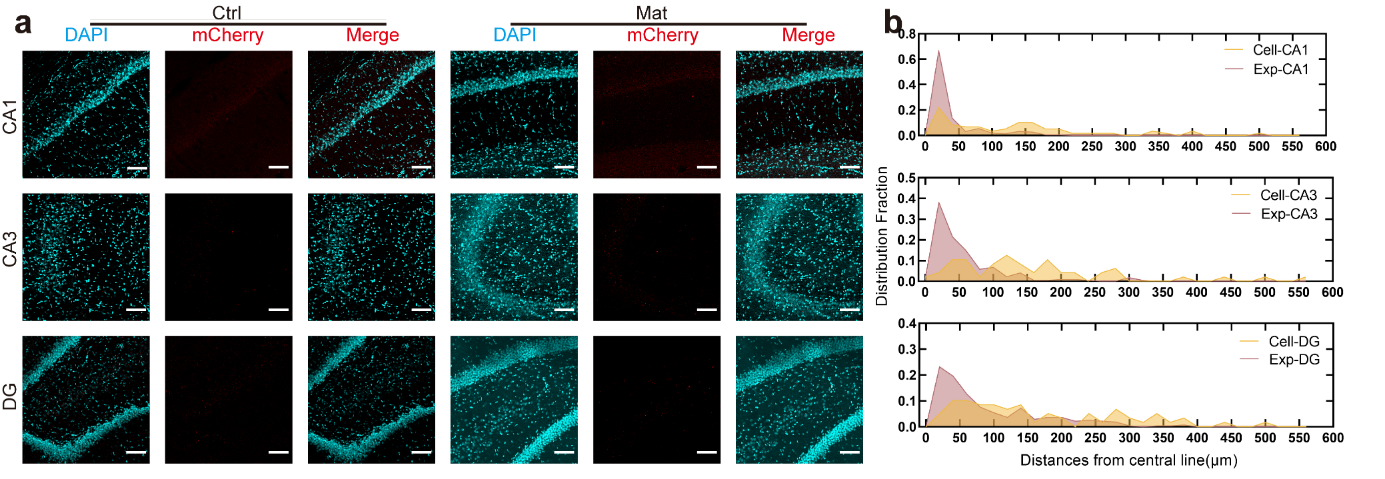


**Figure S6. Transplanted cell retention analysis.** **a,** *In vivo* imaging comparison of Ctrl group and Mat group, confirming signal specificity above background noise. Scale bars: 100 μm. **b,** Spatial distribution of transplanted cells relative to principal cell layers.


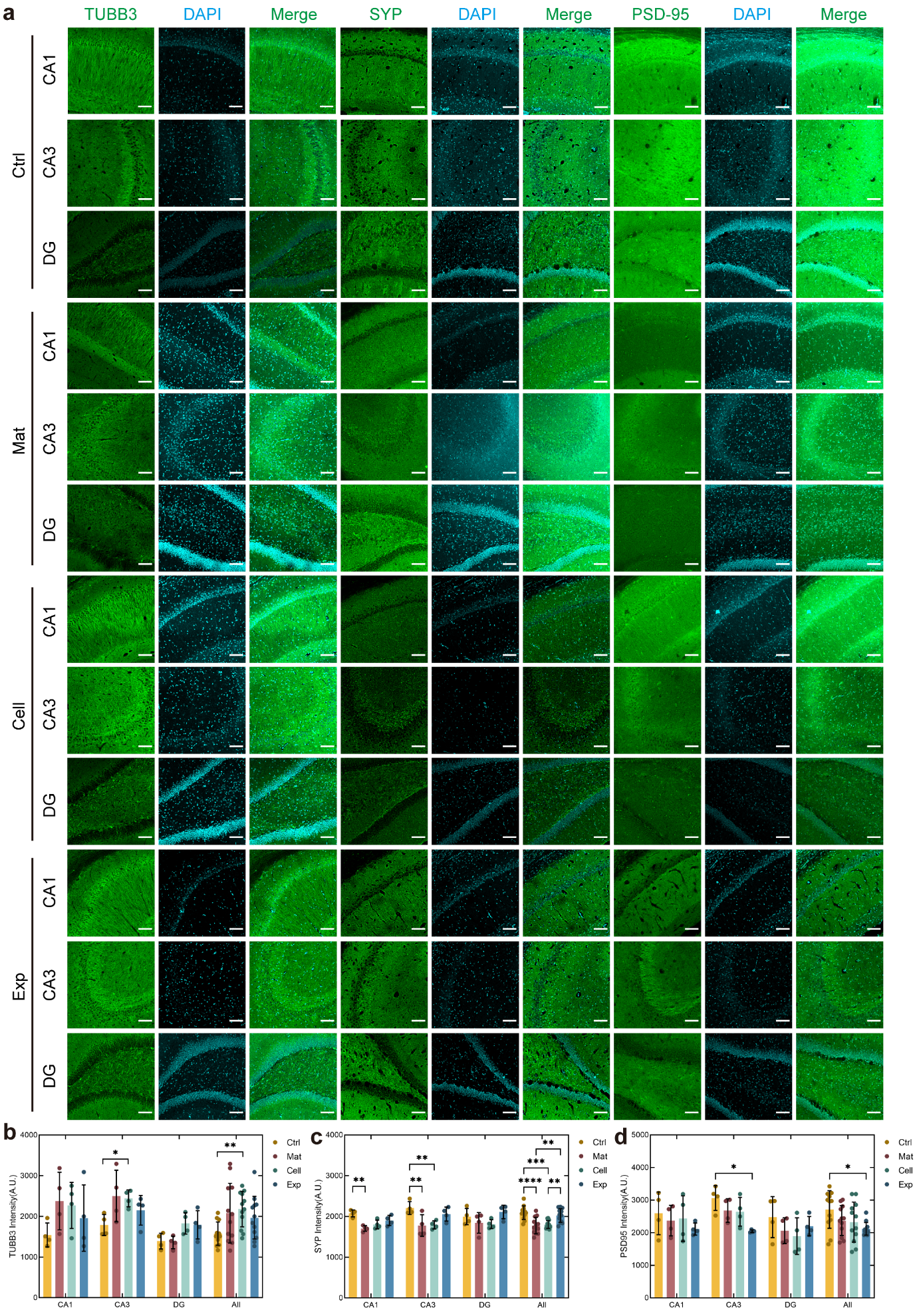


**Figure S8. Staining and analysis of hippocampal neural network function-related proteins in four groups of animals.** **a,** Expression of TUBB3, SYP, and PSD-95 in the CA1, CA3, and DG regions of the hippocampus in four groups of animals. Scale bars: 100 μm. **b-d,** Corresponding fluorescence intensity analysis. Data presented as mean ± SD. Statistical comparisons were performed using two-way ANOVA with Tukey’s post hoc test for multiple comparisons for **c**. Due to heteroscedasticity, two-way Welch’s ANOVA with Games-Howell post hoc test was applied in **b** and **d**. (n = 4, 4, 4, 12)


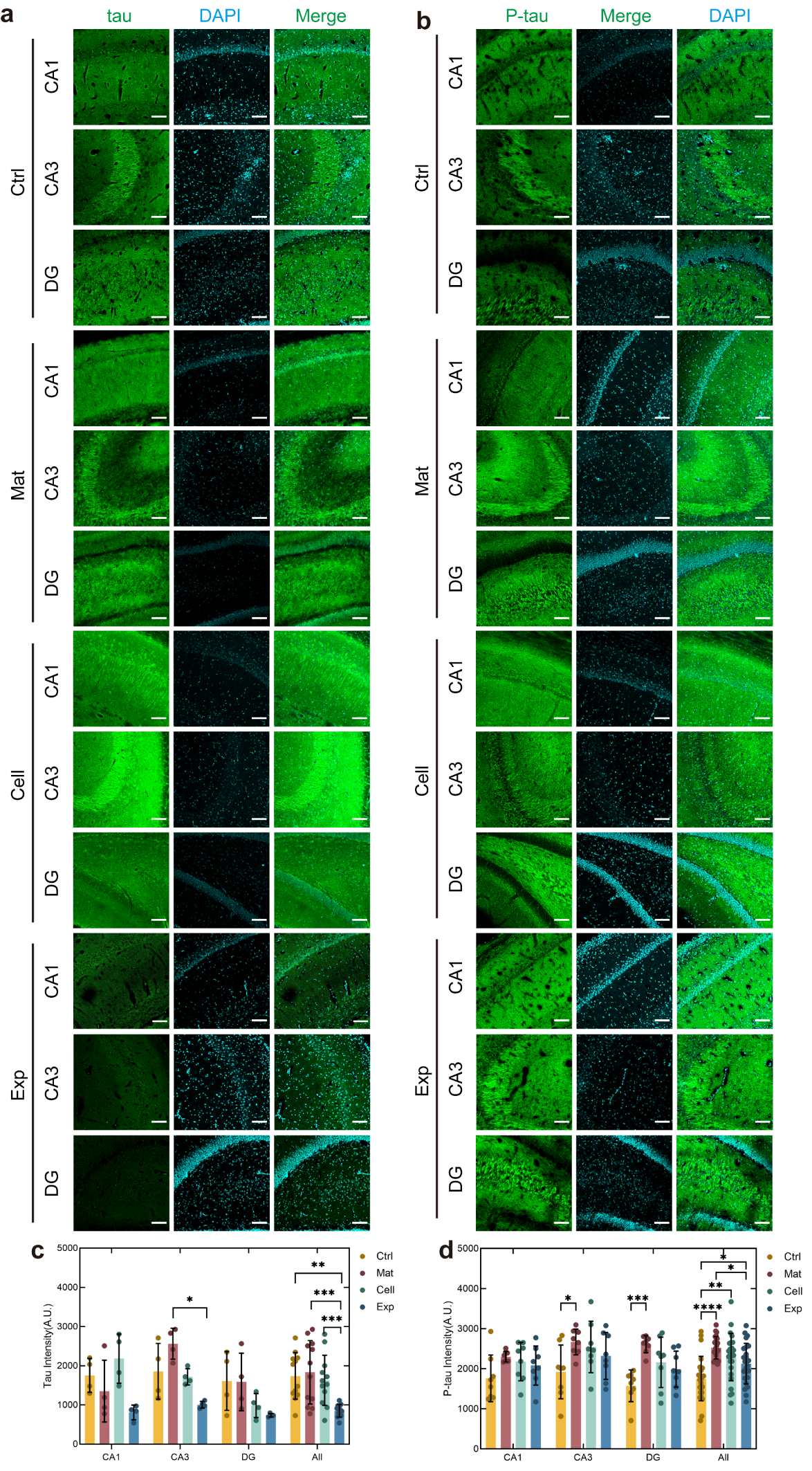


**Figure S9. Staining and analysis of AD tau pathology-related proteins in the hippocampus of four groups of animals. a-b,** Expression of tau and P-tau in the CA1, CA3, and DG regions of the hippocampus in four groups of animals. Scale bars: 100 μm. **c,** Original tau fluorescence intensity analysis. Statistical differences were determined using two-way Welch’s ANOVA followed by Games-Howell post hoc tests. (n = 4, 4, 4, 12) **d,** Original P-tau fluorescence intensity analysis. Statistical differences were determined using two-way ANOVA followed by Tukey's multiple comparisons test. (n = 8, 8, 8, 24). Data presented as mean ± SD. *p < 0.05, **p < 0.01, ***p < 0.001, ****p < 0.0001.


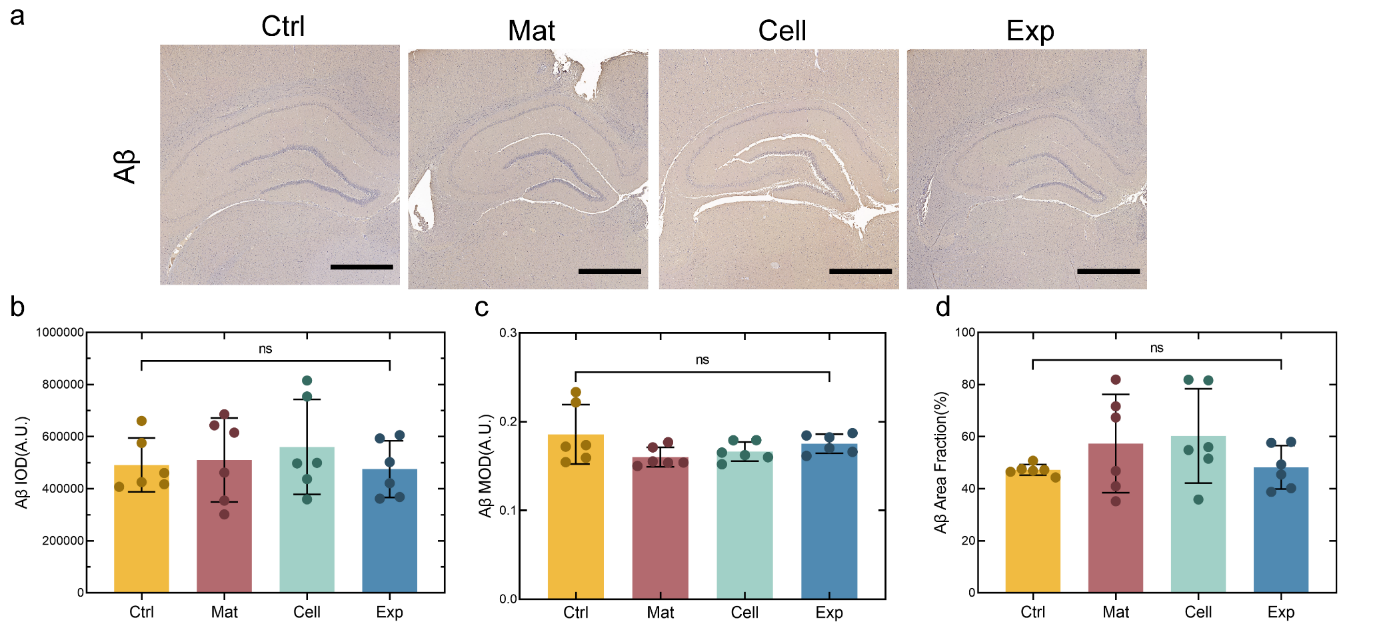


**Figure S10. Aβ IHC results and analysis in the hippocampal region across the four animal groups. a,** Aβ IHC staining results. Scale bar = 1000 μm. **b**, Total optical density analysis. **c**, Mean optical density analysis. **d**, Percent positive area analysis. Data presented as mean ± SD; n = 6. P-values were calculated using Welch's ANOVA test followed by Dunnett’s T3 multiple comparisons test. *p < 0.05, **p < 0.01, ***p < 0.001, ****p < 0.0001.


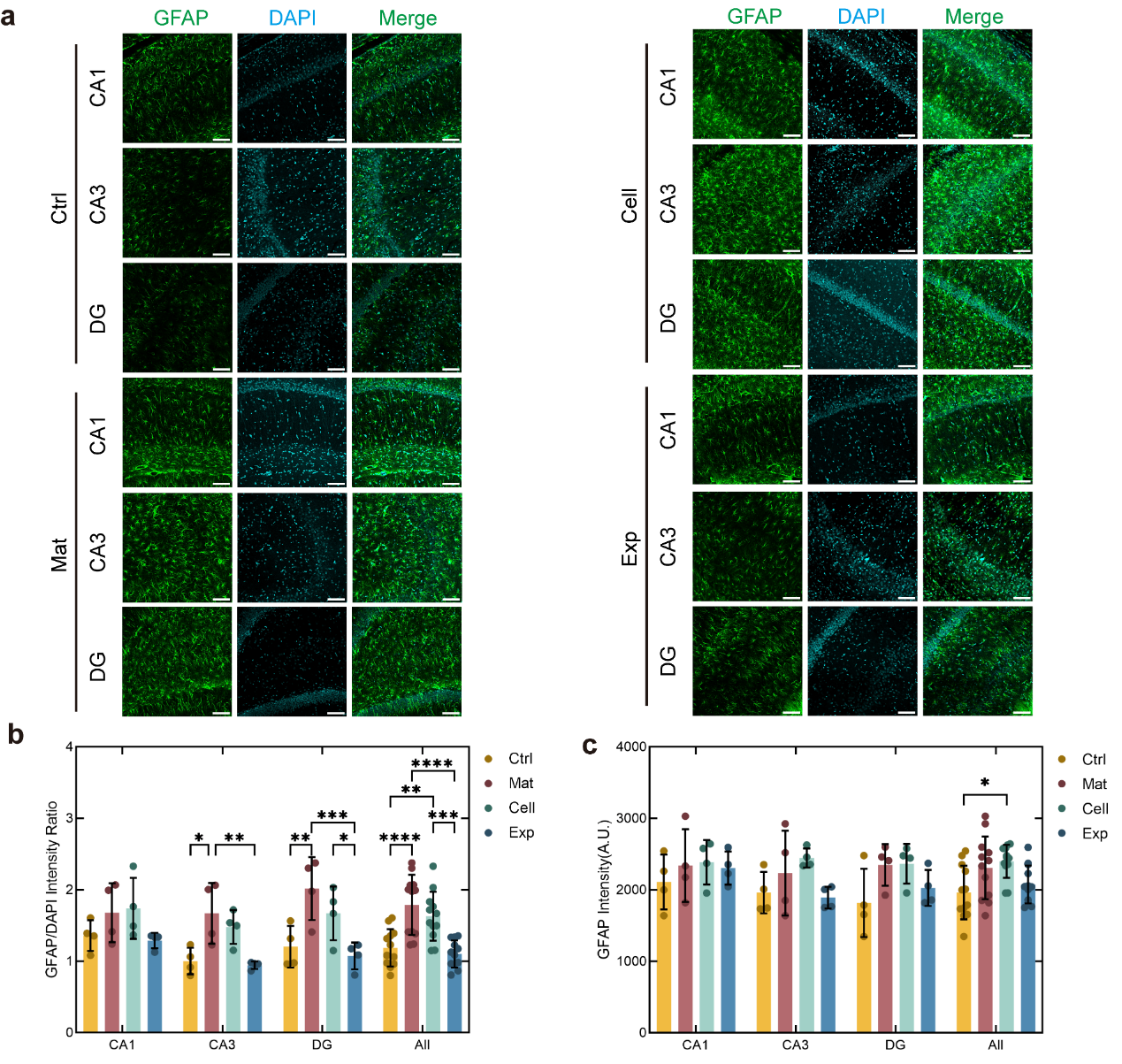


**Figure S11. GFAP immunofluorescence results and analysis in the hippocampal region across the four animal groups. a,** Representative GFAP IF images. Scale bar = 100 μm. **b,** Normalized GFAP fluorescence intensity (ratio of GFAP to DAPI mean fluorescence intensity). **c,** Raw GFAP fluorescence intensity. Data are presented as mean ± SD; n = 4, 4, 4, 12. P-values were calculated using Two-way ANOVA followed by Tukey's multiple comparisons test. *p < 0.05, **p < 0.01, ***p < 0.001, ****p < 0.0001.


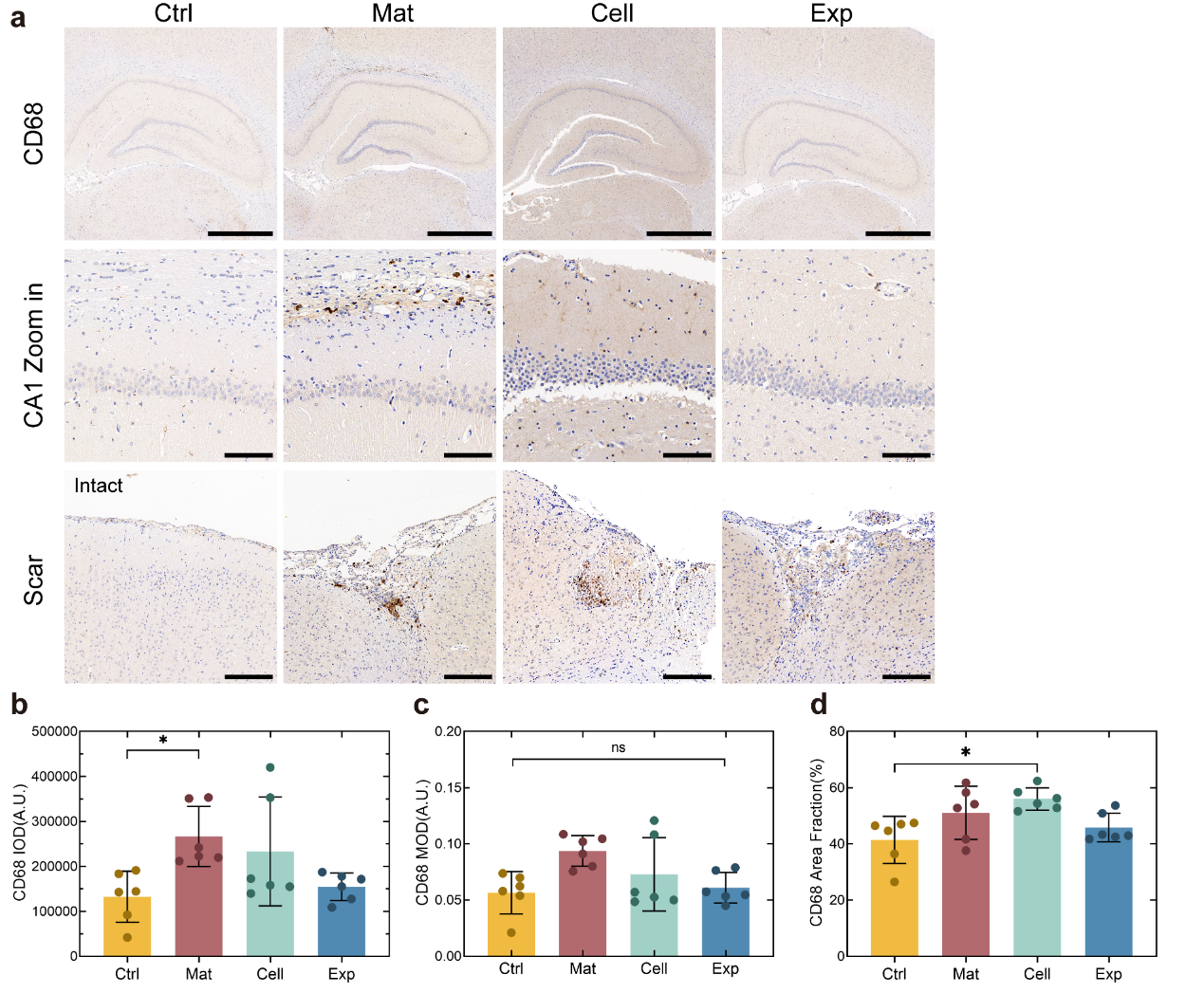


**Figure S12. Immunohistochemical analysis of CD68 expression in the four groups.** **a,** Representative IHC images depicting CD68 expression. Scale bars: 1000 μm (overview of the hippocampus), 100 μm (higher-magnification inset), and 200 μm (scar region). **b-d,** Quantification of the IOD, MOD, and positive area fraction from the hippocampal overviews. Data are presented as mean ± SD; n = 6. P-values were calculated using the Kruskal-Wallis test followed by Dunn's multiple comparisons test. *p < 0.05, **p < 0.01, ***p < 0.001, ****p < 0.0001.


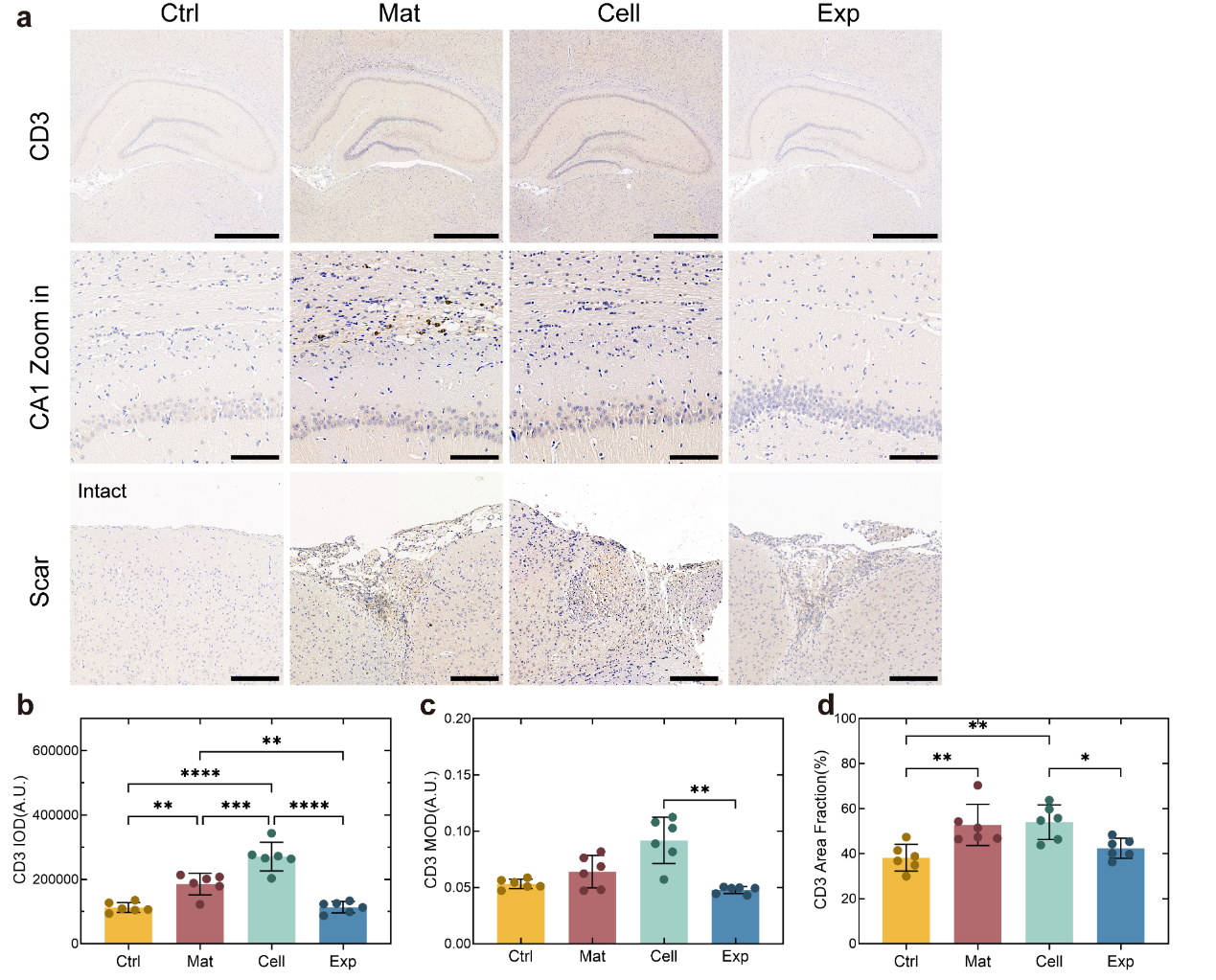


**Figure S13. Immunohistochemical analysis of CD3 expression in the four groups.** **a,** Representative IHC images depicting CD3 expression. Scale bars: 1000 μm (overview of the hippocampus), 100 μm (higher-magnification inset), and 200 μm (scar region). **b-d,** Quantification of the IOD, MOD, and positive area fraction from the hippocampal overviews. Data are presented as mean ± SD; n = 6. P-values in **b** and **d** were calculated using ordinary one-way ANOVA followed by Tukey's multiple comparisons test, while the p-values in **c** was determined by the Kruskal-Wallis test followed by Dunn's multiple comparisons test. *p < 0.05, **p < 0.01, ***p < 0.001, ****p < 0.0001.


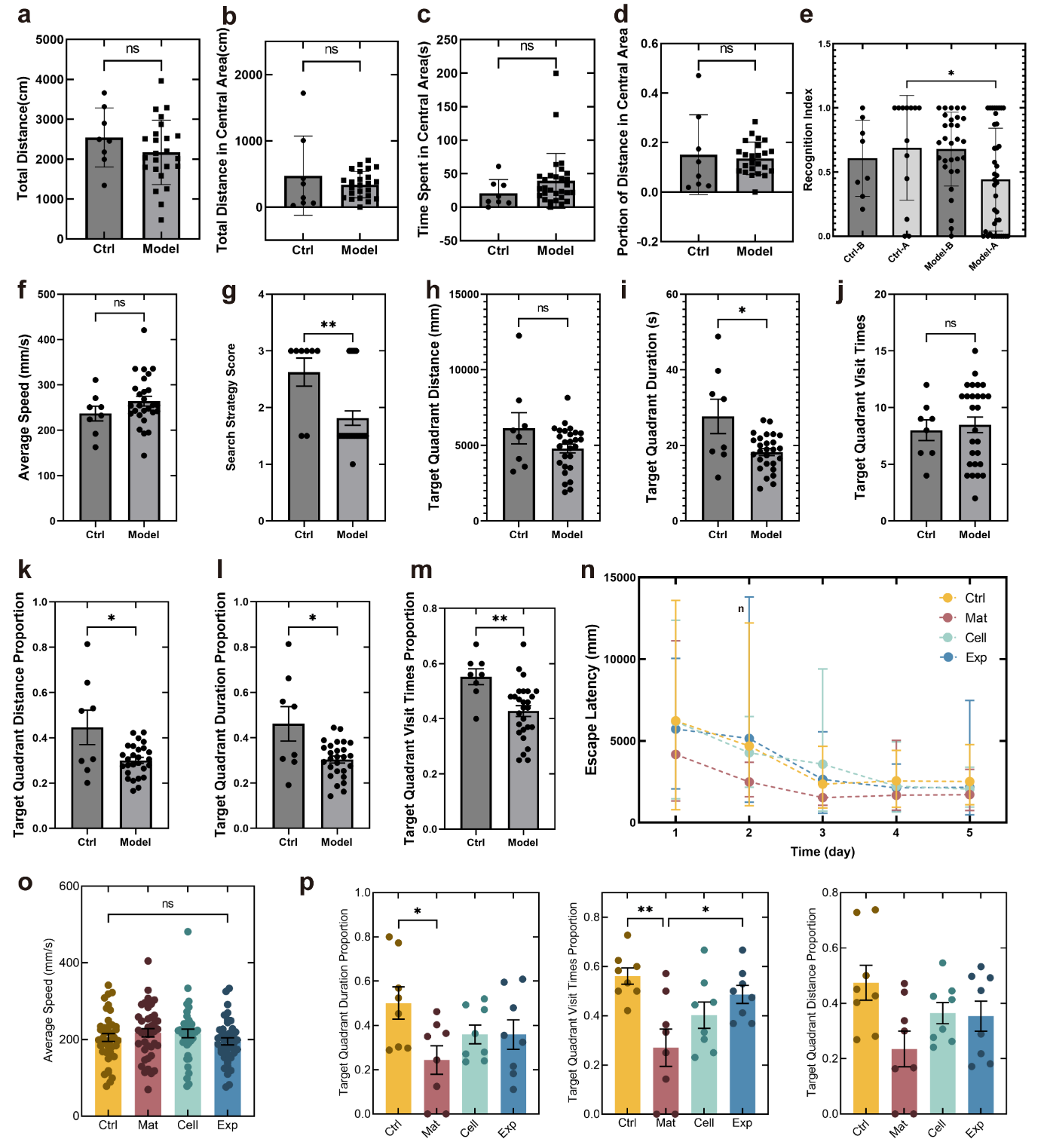


**Figure S14. Behavioral analysis of animal modeling and differential analysis of water maze behavior in four groups of animals. a-d,** Open-field behavioral test performance of control and model group animals after modeling including total distance, central area distance, central area time, and central area distance ratio. (n=8, 24) **e,** Novel object recognition test performance of control and model group animals after modeling. Recognition Index = New object area time/Total time. (n=13, 39) **f-m,** Water maze behavioral test performance of control and model group animals after modeling including the average speed, search pattern score, target quadrant distance, target quadrant time, target quadrant visit times, and the corresponding ratio of the total count. (n=8, 27) **n,** Escape latency during water maze training. (n=8) **o-p,** Water maze behavioral test performance of four groups of animals after transplantation treatment. **o** represented the average swimming speed. (n=40) **p** represented the target quadrant time, target quadrant visit times, and the ratio of target quadrant distance to the corresponding total count. (n=8) For **n**, results presented as mean with range. For other pannels, data presented as mean±SEM. For panels a, f, and m, unpaired t-tests were applied; for panels b, c, d, e, g, and j, the Mann-Whitney test was used; for panels h, i, k, and l, Welch's t-test was employed. Panel o was analyzed using the Kruskal-Wallis test followed by Dunn's multiple comparisons test, and panel p was analyzed using ordinary one-way ANOVA with Tukey's multiple comparisons test. *p < 0.05, **p < 0.01, ***p < 0.001, ****p < 0.0001.
